# Exploring methods to assess environmental health inequalities in health impact assessments of local interventions: a systematic review within the JA PreventNCD project

**DOI:** 10.3389/fpubh.2025.1546394

**Published:** 2025-03-19

**Authors:** Sara Properzi, Angela Andrea Coa, Claudio Fiorilla, Roberto Pasetto

**Affiliations:** ^1^Department of Medicine and Surgery, University of Perugia, Perugia, Italy; ^2^Unit of Environmental and Social Epidemiology, Department of Environment and Health, Istituto Superiore di Sanità, Rome, Italy; ^3^Department of Medical and Surgical Sciences, University of Bologna, Bologna, Italy; ^4^Department of Public Health, “Federico II” University, Naples, Italy; ^5^WHO Collaborating Centre for Environmental Health in Contaminated Sites, Istituto Superiore di Sanità, Rome, Italy

**Keywords:** health impact assessment, inequalities, inequities, vulnerabilities, environmental justice, local intervention, JA PreventNCD

## Abstract

**Background:**

Health Impact Assessment (HIA) procedures can include the assessment of inequalities and inequities associated with the distribution of environmental health risks and benefits, aimed at attenuating the exacerbation of environmental health disparities. This systematic review, conducted as part of the Joint Action Prevent Non-Communicable Diseases initiative, explores methods for assessing health inequalities and equity within HIA frameworks, particularly in local projects affecting the distribution of environmental risks and benefits.

**Methods:**

Adhering to the PRISMA guidelines, a systematic review of the scientific literature was conducted using the MEDLINE/PubMed, Scopus, and Embase databases, searching until March 8, 2024. Furthermore, a grey literature analysis encompassed the Institutional Repository for Information Sharing (IRIS) of the World Health Organization, to identify guidelines and recommendations addressing equity considerations in HIAs. Studies were included based on predefined eligibility criteria if they explored issues related to inequalities, inequities, and vulnerabilities within the context of HIAs. Data extraction focused on methodologies that incorporated equity considerations within the HIA framework, particularly concerning local urban planning initiatives, transport infrastructure, and industrial settings.

**Results:**

A total of 33 studies met the inclusion criteria. Among these, eight documents from the grey literature, identified as guidelines and guidance, underscored the importance of prioritizing equity to ensure that health impacts are addressed fairly across diverse population groups. The remaining 25 peer-reviewed studies employed a combination of quantitative and qualitative methodologies. Quantitative approaches, including exposure-response modeling and Geographic Information System (GIS) mapping, were utilized to evaluate spatial and demographic health disparities. Qualitative methods, such as focus groups, interviews, and participatory tools, provided insights into the lived experiences of vulnerable populations affected by local interventions. Studies addressing urban and transportation planning predominantly emphasized socioeconomic stratification, whereas those focused on industrial settings highlighted occupational hazards and community vulnerabilities.

**Conclusion:**

This review highlights the diverse and fragmented approaches used to address health inequalities and equity in HIA. It underscores the need for interdisciplinary and systematic methodologies that integrate quantitative and qualitative perspectives, ensuring equity remains a central consideration in policymaking and project implementation. Finally, it proposes a practical framework for integrating equity into HIA.

## 1 Introduction

The exacerbation of the socioeconomic gap between privileged segments of the population and the majority of citizens arises from a multifaceted interplay among social policies, governmental programs, disadvantageous economic decisions, and ineffective governance (1). This disparity is also evident regarding access to ecosystem resources, which comprise the functions and ecological processes that directly or indirectly impact human well-being (2, 3). It is important to highlight that human well-being is influenced by the complex interaction among various types of capital. This encompasses natural capital and its associated resources, human and social capital (4), and built capital, which comprises physical infrastructure such as buildings, machinery, and transportation systems, along with other anthropogenic services (5). Furthermore, providing ecosystem resources is intrinsically linked to the conditions of the surrounding ecosystem (6). The ecosystem is deemed healthy when it demonstrates stability and sustainability over time, maintains its structure and autonomy, and exhibits resilience to external stresses (7, 8).

Health Impact Assessment (HIA) has been increasingly advocated as an essential tool for safeguarding public health (9). Characterized by a prospective multidisciplinary approach, HIA aims to identify the potential consequences, both negative and positive, of interventions, programs, or projects on population health, as well as the distribution of these effects within the population itself (10). The primary objective of HIA is to preserve and promote health while concurrently mitigating potential harms (11, 12). Therefore, it plays a crucial role in guiding policies and interventions (13) aimed at protecting and improving public health.

In the twenty-first century, the practice of HIA faces increasingly complex challenges, including the imperative recognition of health outcomes as emergent from a multifaceted interplay of proximal and distal determinants, encompassing ecological and planetary dimensions (14).

In HIA, it is of paramount importance to pay attention to the differential distribution of effects on distinct population groups, with particular regard to so-called “vulnerable populations,” including communities that exhibit widespread fragility overall, or vulnerable population groups such as the older adult, children, or those with specific characteristics, such as ethnic minorities or socioeconomically disadvantaged groups (15–17). Socioeconomic disadvantage is frequently observed in communities near contaminated areas (18). Moreover, inhabiting areas near contaminated sites, often in densely populated urban areas for historical reasons, is associated with severe health impacts and decreased life expectancy and quality of life (19).

Through the stages of Screening, Scoping, Assessment, Reporting, and Monitoring (20), the HIA process incorporates an appraisal of community vulnerability and delineation of demographic cohorts within the populace potentially susceptible to disproportionate exposure to adverse consequences resulting from novel developments in their area. This comprehensive approach permits the formulation of recommendations directed towards guiding decisions in alignment with principles of equity, thereby ensuring that policies and interventions can facilitate an equitable apportionment of health risks and benefits while mitigating the exacerbation of health disparities.

Equity has been identified as a core value in HIA practices since 1999 by the World Health Organization (10), and this concept is increasingly emphasized in the scientific literature (20, 21). Therefore, it is crucial to ensure that inequalities and equity considerations are substantially incorporated within HIA procedures to prevent the perpetuation or even amplification of existing health disparities.

Many HIAs tend to adopt an approach that considers a single “social determinant of health” (22) to assess the impacts of specific projects, despite the growing recognition that health equity can be influenced by the combination of a single project with multiple factors (23).

Addressing health inequalities in HIA related to new projects and interventions that influence the distribution of environmental risks and benefits is particularly critical in areas with a long history of environmental pressures. The communities living in such areas, often overburdened by extensive and long-lasting industrial contamination, face a strong correlation between their socioeconomic conditions and environmental quality, which in turn impacts their health (24). A pilot action within the work package on social inequalities of the European Joint Action Prevent Non-communicable Diseases (25) is developing an approach to prevent non-communicable diseases in such communities while promoting environmental justice.

This systematic review aims to explore both peer-reviewed scientific literature and grey literature to understand how inequalities and equity considerations have been addressed within HIA procedures concerning projects that may influence variations in the distribution of environmental risks and benefits within a territory. Based on the literature findings, recommendations will be provided for assessing inequalities and equity in HIA practice.

## 2 Materials and methods

### 2.1 Search strategy

We systematically reviewed the scientific literature according to PRISMA Guidelines (26), utilizing the MEDLINE/PubMed, Scopus, and Embase databases. The search was conducted until March 8, 2024, without any temporal restrictions. Initially, the search commenced on MEDLINE, after which an appropriate syntax was established for the remaining databases. Search terms encompassed assessing health impact, inequalities and inequities, vulnerability, environmental justice, industrial facilities, transportation infrastructures, and urban planning. The comprehensive search strategy is depicted in Table 1 in Supplementary File 1.

Through the methodological approach of “Snowballing” (27), studies deemed relevant to our objectives were identified to ensure comprehensive and inclusive coverage of the literature. Furthermore, a grey literature analysis was conducted to identify guidelines and methods for HIA to integrate into the systematic review. This process involved examining the content of the World Health Organization’s Institutional Repository for Information Sharing (IRIS) (28), utilizing the MeSH term “health impact assessment” as a filtering criterion. The protocol for this systematic review has been registered and is available on PROSPERO (CRD42024522697; http://www.crd.york.ac.uk/PROSPERO).

### 2.2 Eligibility criteria

The included studies met the following criteria: publications in the English language; experimental and observational studies examining inequalities, inequities, and vulnerabilities in the context of HIA; HIA practices applied to various settings (industrial facilities, transportation infrastructures, and urban planning) to assess how inequalities, inequities, and vulnerabilities were analyzed; studies reporting methodologies used and focusing on assessment at local or area level rather than global scale.

In the equity context for the terms *inequality, inequity*, and *vulnerability*, we have adhered to the definitions provided by the Oxford Reference, respectively, for Health Inequalities (29), Health Inequity (30), and Vulnerability (31). Oxford Reference defines Health Inequalities as “Differences in health status or in the distribution of health determinants between different population groups. Some are attributable to biological variations or free choice, and others to the external environment and social conditions outside the control of individuals. In the latter case, they may be unnecessary and avoidable as well as unjust and unfair, and cause or reflect health inequity” (29); Health Inequities as “Systematic health inequalities that are a result of modifiable social and economic policies and practices that create barriers to opportunity” (30); Vulnerability as “A position of relative disadvantage; e.g., owing to impaired nutrition, cognition, or socioeconomic position. The extent to which a person, population, or ecosystem is unable or unlikely to respond or adapt to threats” (31).

Variables identified through the PROGRESS plus acronym (32) were also considered in the equity dimension. This tool is employed to analyze characteristics influencing opportunities and outcomes in the healthcare context. PROGRESS components include place of residence, race/ethnicity/culture/language, occupation, gender/sex, religion, education, socioeconomic status, and social capital (32). The “Plus” component extends to personal characteristics related to discrimination, such as age and disability, relational dynamics like parental smoking or school exclusion, as well as temporal relationships such as hospital discharge period or access to temporary care. To ensure coherence and alignment with the focus of the study, studies that were deemed irrelevant to the research topic, those lacking complete availability, and duplicates were systematically excluded.

### 2.3 Study selection

**Figure 1:**
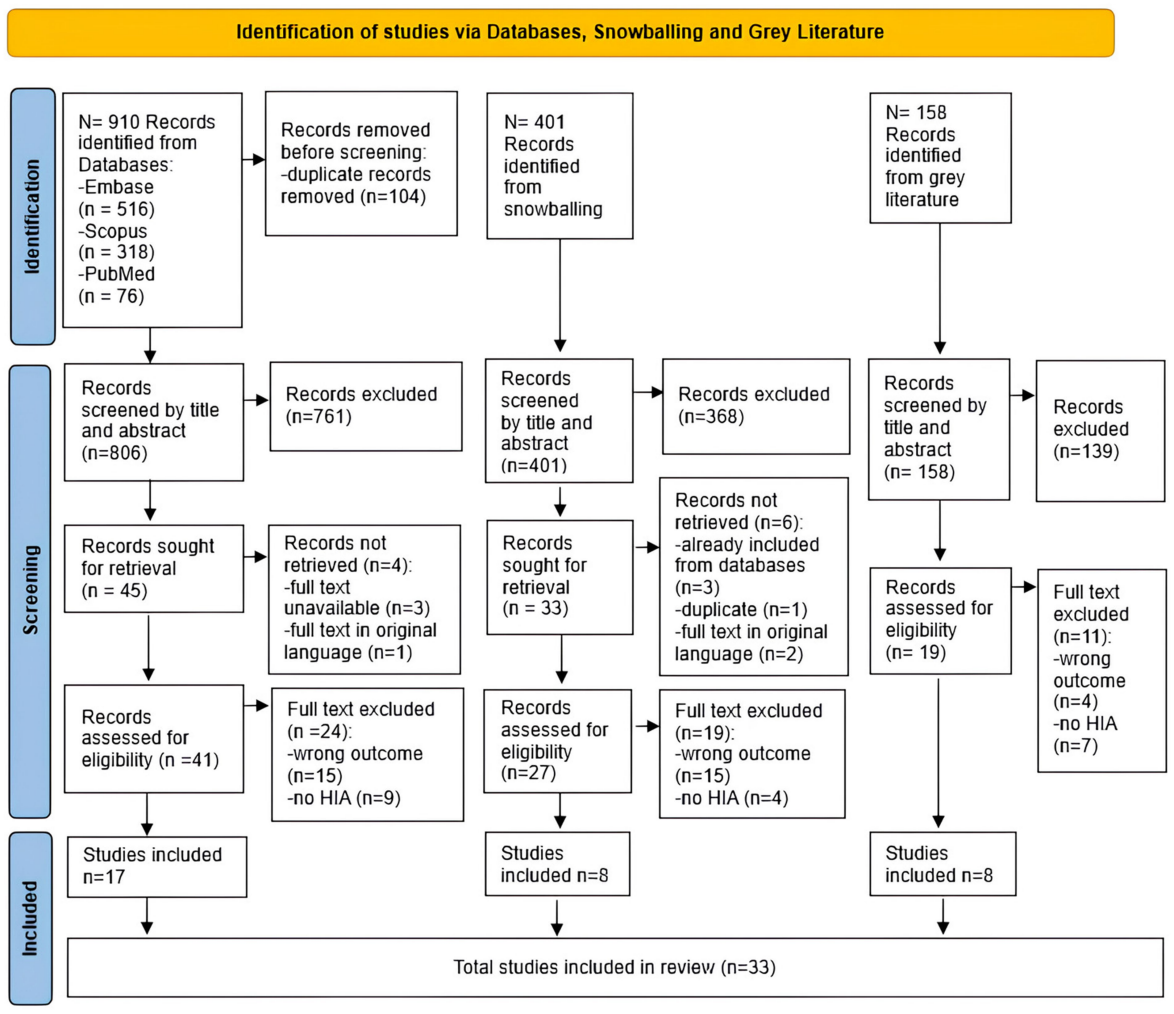
PRISMA flow chart.

The first author (SP) imported the peer-reviewed literature into the Rayyan online platform (33). After the elimination of duplicate entries, three reviewers (SP, AAC, and CF) independently screened articles, initially based on title and abstract, then by full-text examination, to ascertain eligibility for final inclusion. Discrepancies during screening were resolved through consensus or consultation with a fourth reviewer (RP). Ineligible sources were systematically recorded at this stage, along with a rationale for their exclusion (Figure 1).

Articles identified through the snowballing technique (27), based on the references of the included studies, and deemed relevant to the review objectives, were incorporated into the final analysis (Figure 1).

Guidelines available on the subject, derived from research in the World Health Organization’s Institutional Repository for Information Sharing (IRIS) (28), using the MeSH term “health impact assessment” as a filtering criterion, underwent full-text screening by the three reviewers (SP, AAC, and CF) independently. Guidelines and guidances judged relevant were included in the review.

The subsequent phase involved data extraction and cumulative assessment by the reviewers.

### 2.4 Methodological quality assessment

The methodological quality assessment was conducted using the Wales Health Impact Assessment Quality Assurance Review Framework (34), a standardized form of quality assurance for HIAs. This tool enabled an evaluation of the HIA process by focusing on key criteria such as stakeholder engagement, the integration of evidence, and the thoroughness of impact evaluations. The Wales Health Impact Assessment Quality Assurance Review Framework was originally designed to assess the comprehensiveness of the HIA approach rather than the quality of the study’s outcomes. Moreover, its validity is intended to apply on a case-by-case basis, as it was not developed as a comparative tool for evaluating multiple studies against one another.

The tool consists of two appendices: a primary appendix known as the Review Criteria Matrix, with 43 questions, and a corresponding supplementary appendix referred to as the Explanatory Notes. For each question there is an answer that indicates the quality, such as “good,” “requires strengthening” or “insufficient.”

Our objective in using the tool was to determine how many studies addressed equity-related questions and how they did so, while also gaining a broader understanding of the quality of HIA approaches in the examined studies. Since the tool does not define specific ranges for quality but relies on the knowledge and experience of researchers in HIA to assess the quality level, we limited ourselves to applying the tool and reporting the number of responses classified as “good,” “requires strengthening,” or “insufficient” (Supplementary File 2b). To ensure consistency, experienced researchers independently conducted assessments, and disagreements were resolved through collaborative discussions, guided by the framework’s notes and the team’s collective expertise.

Further details on the tool used for Methodological quality assessment can be found in Supplementary File 2a.

### 2.5 Data extraction

Systematic extraction of relevant information from each article was conducted using a standardized data extraction form (Microsoft Excel 2019, Microsoft Corp). Extracted data included: first author’s name, year of publication, application site of HIA or site of the relevance of identified guidelines, type of opera/interventions (facilities/transport/infrastructure/urban planning/industrial mine), type of assessment (access, exposure, proximity, health effects, use), methodology used for assessing inequalities and considering equity dimension, and main findings.

The data extraction process was performed by three independent reviewers (SP, AAC, and CF). Discrepancies or divergences in data extraction were resolved through in-depth discussion, consensus-building, or consultation with a fourth reviewer (RP).

### 2.6 Data synthesis

Within the scope of this systematic review, a narrative approach was employed to succinctly summarize the findings of both studies and guidelines, considering qualitative and quantitative assessments.

Evidence derived from HIA procedures and guidelines was analyzed separately from findings resulting from the application of HIA to specific cases. Additionally, data were stratified based on the nature of interventions to facilitate comprehension and comparison across studies.

## 3 Results

### 3.1 Study selection

The initial exploration across three electronic databases (Embase, Scopus, and PubMed) yielded a total of 910 studies. After the removal of duplicates (104) and the exclusion by title and abstract (761), a comprehensive evaluation of the full texts of 45 remaining studies was undertaken to determine their potential inclusion. Regrettably, four articles were not retrieved, either because the full text was missing or because the full text was only available in the original language, and 24 studies were excluded as they did not meet the inclusion criteria (Tables 2, 3 in Supplementary File 1). Ultimately, a selection of 17 articles (35–51), comprising 8 field studies (35–39, 42–44), and 9 guidance (40, 41, 45–51) were included in the review.

Through the snowballing technique, of the 401 records initially identified from the references of included studies, 368 were excluded based on title and abstract screening. The full text of 33 studies was assessed. Of these, 6 studies were excluded for the following reasons: 3 were already incorporated into the evaluation of peer-reviewed literature databases, 1 was identified as a duplicate, and 2 were excluded because the full text was available exclusively in the original language (Table 4 in Supplementary File 1). A total of 27 studies were assessed in full text; 19 full-text studies were excluded from the analysis, with 15 excluded because they did not meet our inclusion criteria, specifically the evaluation of inequalities, inequities, and vulnerabilities within the context of HIA (wrong outcomes) and 4 excluded because they did not focus on HIA (Table 5 in Supplementary File 1), resulting in the inclusion of 8 studies (52–59), of which 5 field studies (52–54, 56, 58), and 3 guidance (55, 57, 59).

The review of grey literature on WHO’s IRIS repository yielded 158 records and 139 were excluded based on title and abstract screening. A full-text evaluation was conducted for 19 documents, excluding 11 of them: 4 due to wrong outcomes and 7 not addressing HIA, which subsequently led to the inclusion of 8 full-text records (60–67) (Table 6 in Supplementary File 1).

Studies categorized as “no HIA” were excluded if, despite addressing inequalities, inequities, and vulnerabilities, they did not represent an HIA process or were not situated within an HIA framework.

The accompanying flow chart comprehensively depicts the study selection process (Figure 1). A comprehensive list of excluded sources from databases and snowballing results has been compiled and is available for reference (Supplementary File 1).

### 3.2 Methodological quality assessment

The application of the *Wales Health Impact Assessment Quality Assurance Review Framework* (34) revealed mixed results regarding the quality of the assessed HIAs.

We identified 3 studies that met the quality threshold we defined as “qualitatively good” (43, 46, 48). Of these, one is a field study (43) and 2 are guidance documents (46, 48). Among these, only 2 studies (43, 46)—1 field study (43) and 1 guidance document (46)—reported a good qualitative assessment for the direct question on equity (Question 6.1 Supplementary File 2b). Furthermore, these 2 studies (43, 46), provided a good qualitative assessment for all indirect questions addressing the equity dimension (Question 4.7, 6.2, 6.3, 6.4, 6.5 Supplementary File 2b).

It emerges that studies evaluated as “qualitatively good” in our assessment adequately integrated the equity dimension into the evaluation of health impact. Conversely, studies that fell slightly below the threshold of good quality (under 26 answers classified as “Good) (40, 55, 57) partially addressed the equity dimension. These studies reported “required strength” for the direct question on equity (Question 6.1 Supplementary File 2b), while their responses to indirect equity-related questions exhibited high variability (Question 4.7, 6.2, 6.3, 6.4, 6.5 Supplementary File 2b).

Nevertheless, the three studies classified as “qualitatively good” (43, 46, 48) failed to adequately investigate the overall health impact, encompassing both physical and mental health (Questions 2.4 and 2.5 Supplementary File 2b). They also did not address an effective monitoring process or the implementation of good practices for the future, nor did they propose a dissemination and sharing of the results (Questions 5.5, 5.6, and 5.13 Supplementary File 2b). Only two studies (39, 40) provided “qualitatively good” responses to the two questions regarding the comprehensive evaluation of health, both physical and mental (Questions 2.4 and 2.5 Supplementary File 2b), while no study sufficiently developed monitoring processes, future proposals, or strategies for the dissemination of results (Questions 5.5, 5.6, and 5.13 Supplementary File 2b).

Further details on methodological quality assessment can be found in Supplementary File 2b.

### 3.3 Study characteristics

Among the included studies, regardless of whether they were field studies or guidance, considering both those derived from peer-reviewed literature and snowballing, all were published from 2002 to 2023. Nineteen studies were conducted in a single country (35, 36, 38–43, 46–51, 53–57), while six studies were carried out across multiple countries (37, 44, 45, 52, 58, 59). Specifically, for the sake of convenience, we have labeled all studies conducted in any single state of the United States as “USA studies.” Similarly, those conducted in any single state of the United Kingdom have been categorized as “UK studies.”

A total of 11 (35–38, 41, 42, 49, 51, 55, 56, 58) studies examined inequity, inequality, and vulnerability within urban and transportation planning. Among these, 3 were conducted in the UK (36, 49, 51), 2 in Spain (55, 56), 1 in the USA (42), 1 in California and Canada (58), 1 in Brazil (35), 1 in Austria (38), and 1 in China (41). Only one study was performed across six cities in Bulgaria, Finland, Ireland, Estonia, Sweden, and France (37).

Eight studies (39, 40, 43, 46–48, 50, 57) evaluated inequity, inequality, and vulnerability in the urban planning framework. Two of them were carried out in Spain (40, 43), 2 in the USA (46, 57), 2 in the UK (47, 50), and 1, respectively, in Canada (39) and Korea (48).

Furthermore, 6 studies (44, 45, 52–54, 59) specifically evaluated inequity, inequality, and vulnerability in the industrial mining site. Specifically, 2 studies focused on Burkina Faso, Ghana, Mozambique, and Tanzania (45, 59), 1 study examined Burkina Faso, Mozambique, and Tanzania (44), and another explored Australia, South Africa, and Namibia (52). The remaining 2 studies were confined to single countries, with 1 conducted in Tanzania (53), and 1 in Zambia (54).

**Figure 2:**
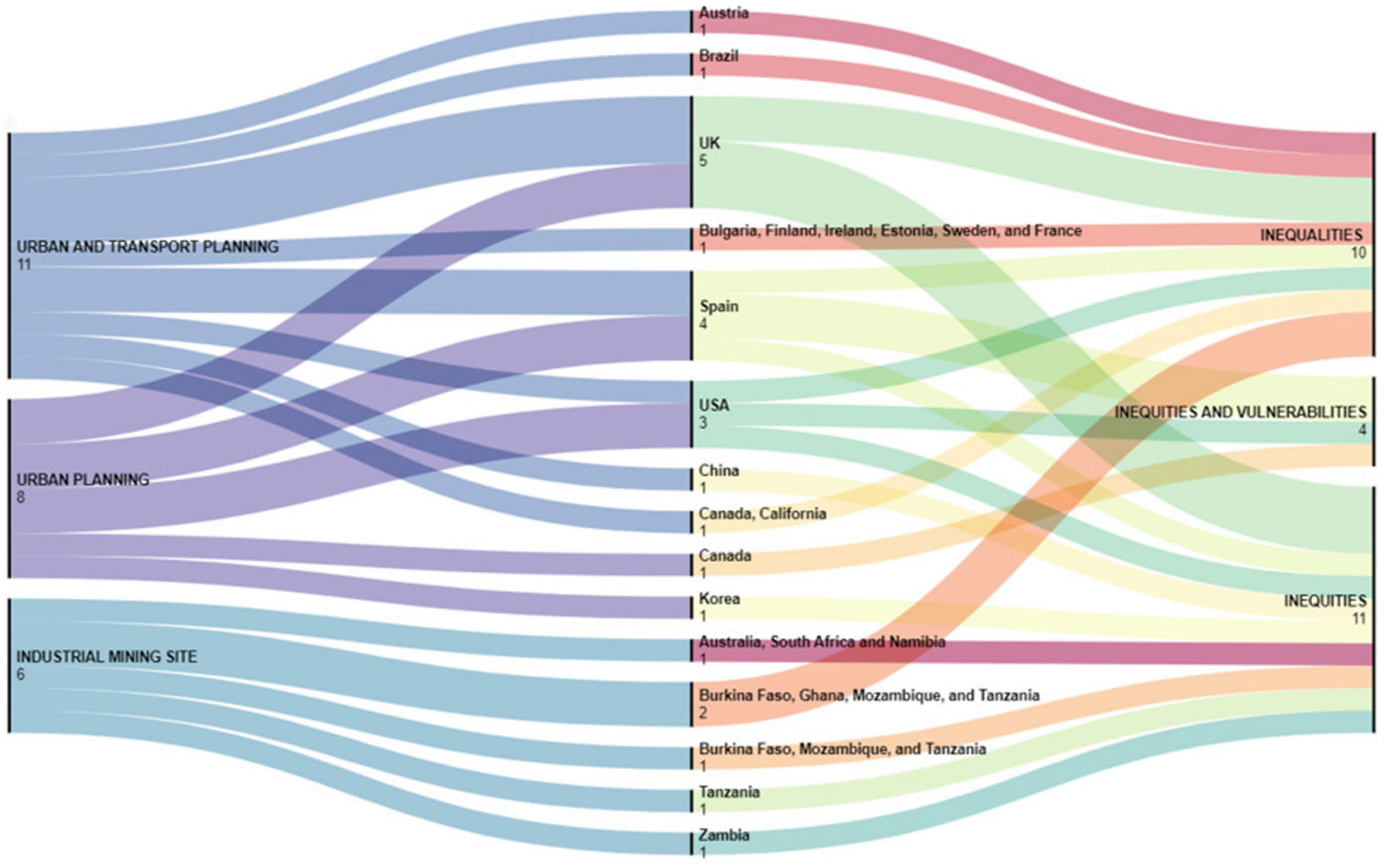
Opera, country, and inequalities, inequities, inequities, and vulnerabilities evaluated in studies from literature peer-reviewed (https://app.rawgraphs.io/).

All the information is represented in Figure 2. Furthermore, Tables 1, 2 provide an overview of the inequalities, inequities, and vulnerabilities addressed, as well as the setting and the methods employed to assess them.

**Table 1:** Methods to assess inequalities, inequities, and vulnerabilities from field studies.

References	Inequities, inequalities, and vulnerabilities evaluated	Opera Setting	Methods
Barboza et al. (35)	Inequalities Different mortality impacts of exposure by socioeconomic factors.	Urban and Transport Planning related exposure (green space, air pollution and heat)	Comparison of exposure levels and counterfactual scenarios
Mueller et al. (36)	Inequalities Different mortality impacts of exposure by socioeconomic factors.	Urban and Transport Planning related exposure (Physic Activity (PA), air and noise pollution, and green space)	Comparison of exposure levels and counterfactual scenarios
Barboza et al. (37)	Inequalities Different mortality impacts of exposure by socioeconomic factors.	Urban and Transport Planning related exposure (green space and air pollution)	Comparison of exposure levels and counterfactual scenarios
Khomenko et al. (38)	Inequalities Different mortality impacts of exposure by socioeconomic factors.	Urban and Transport Planning related exposures (PA, air and noise pollution, green space and heat)	Comparison of exposure levels and counterfactual scenarios
Iungman et al. (56)	Inequalities Different mortality impacts of exposure by socioeconomic factors	Urban and Transport Planning related exposures (PA, air and noise pollution, green space and heat)	Comparison of exposure levels and counterfactual scenarios
Iroz-Elardo et al. (58)	Inequalities Potential health benefits for impacted communities; consideration of community perspectives regarding safety and social cohesion, employment, and health	Urban and Transport Planning related exposure (Clark County Bicycle and Pedestrian HIA, Lake Merritt BART Station Area Plan HIA, and the I-710 Corridor HIA)	Document review Semi-structured interviews Qualitative content analysis of HIA
Sampson et al. (42)	Inequities and Vulnerabilities Different health impacts on vulnerable residents in the survey area; neighborhood perceptions, and intention to move from the area	Urban and Transport Planning related exposure (Gordie Howe International Bridge)	Cross-sectional surveys
Serrano et al. (43)	Inequities and Vulnerabilities Potential impacts perceived by stakeholders and community groups on the urban environment, health/quality of life and health inequality issues, according to gender, age, and socioeconomic factors.	Urban Planning related exposure (New fish market (NFM) and redevelopment of the La Herrera (LH) zone)	Scientific literature review In-depth informant interviews Focus groups Quota sampling Telephone survey
Anderson et al. (39)	Inequities and Vulnerabilities Potential unintended impacts of green infrastructure on vulnerable populations (older adult, children, individuals of lower SES, individuals with chronic illness or disability)	Urban Planning related exposure (green space, and agriculture and tree-based intercropping systems)	Key informant Interviews Surveys
Knoblauch et al. (54)	Inequities Different health determinants and outcomes in impacted communities versus the comparison communities	Industrial Mining Site	Questionnaire Survey Biomedical and Parasitic Infection Assessment
Leuenberger et al. (44)	Inequities Perceived inequities related to health determinants in interested communities	Industrial Mining Site	Transect Walk Focus Group Discussions (FGDs)
Leuenberger et al. (53)	Inequities Perceived inequities related to health determinants in subpopulation groups (men, women, adolescent boys and girls, and children)	Industrial Mining Site	Transect Walk FDGs
Leuenberger et al. (43)	Inequalities Potential perceived health impacts by the surrounding communities	Industrial Mining Sites	Transect walk FGDs
Anaf et al. (52)	Inequities Positive and negative impacts on workers and local communities related to political and business practices, workforce and working conditions, social conditions, environmental conditions, and economic conditions	Industrial Mining Sites	Media and document analysis, and company literature analysis Semi-structured interviews Key informants meeting

**Table 2:** Methods to assess inequalities, inequities, and vulnerabilities from peer-reviewed guidance.

References	Inequities, inequalities, and vulnerabilities evaluated	Opera Setting	Methods
Gorman et al. (49)	Inequalities Potential different impacts on deprived groups (young families, adolescents, older adult, working people, and unemployed)	Urban and Transport Planning related exposure (Transport planning)	Scientific literature review Policies analysis Key informants meeting
Lester et al. (51)	Inequities Potential different impact on groups who were already disadvantaged (families without car)	Urban and Transport Planning related exposure (Proposed road; the area also includes a country park development)	Discussion meetings
Bacigalupe et al. (55)	Inequities and Vulnerabilities Potential differences in accessibility to Lifts/roads, to parks for vulnerable (older adult, disabled, people without parents)	Urban and Transport Planning related exposure (Integral Regeneration Plan (IRP) of Uretamendi-Betolaza and Circunvalación (UBC))	Scientific literature review Key informants interview Discussion groups
Ge et al. (41)	Inequities Potential impacts in low-income neighborhoods related to public facilities, road transportation, and land use	Urban Planning related exposure (public facilities, road transportation and land use)	Scientific literature review Health determinants analysis relating to socioeconomic status
Richardson et al. (47)	Inequities Potential different impacts of energetic transition on vulnerable groups (low-income households, households in rented houses)	Urban Planning related exposure (Transition Together/Transition Streets (TT/TS) projects)	Documentary evidence Key informants meeting
Kang et al. (48)	Inequities Potential differences in access and use among the vulnerable populations (disabled, lower-income people, and older adult)	Urban Planning related exposure (Aegi-Neung Waterside Park)	Scientific literature review Stakeholders’ workshop
Barnes et al. (50)	Inequalities Potential different impacts on health determinants	Urban Planning related exposure {Regeneration initiatives [New Deal for Communities (NDC)], Single Regeneration Budget (SRB)}	Scientific literature review HIA methods explicitly assess existing health inequalities and the distribution of the potential impacts.
Harris et al. (57)	Inequalities Potential different impacts on health indicators	Urban Planning related exposure (General Plan Update (GPU) to guide future building and growth)	Focus groups
Palència et al. (40)	Inequities Potential impacts perceived related to mental health and health determinants, according to socioeconomic factors	Urban Planning related exposure (Superblocks)	Pre-post health survey Pre-post environmental measurements, environmental walkability measures, use of public space and PA, traffic injury measures Guerrilla ethnography Focus groups
Baskin-Graves et al. (46)	Inequities Potential different impacts on vulnerable populations living near the area or working in the area	Urban Planning related exposure (chicken processing plant)	Resident input and expert interviews Sociodemographic and health data analysis GIS Mapping of environmental hazards
Farnham et al. (59)	Inequalities Potential different impacts on health determinants	Industrial Mining Sites	Key informant interviews and stakeholder meetings with policymakers In-depth interviews and FDGs with impacted communities Semi-structured exit interviews with healthcare providers Analysis of population data and key informant interviews

All inclusions resulting from grey literature (60–67) were published between 2014 and 2023 and were referred to multiple countries, being guidance and guidelines for the European Community. The included texts emphasize the environmental and health risks inherent to urban planning while underscoring the importance of empowerment methodologies to integrate health considerations into environmental and strategic assessments effectively. They further stress the need to prioritize equity, ensuring that health impacts are addressed equitably across diverse population groups.

#### 3.3.1 Opera and infrastructure characteristics

Within the context of urban and transport planning, 5 studies considered basal exposure levels to air (35–38, 56) and noise pollution (36, 38, 56), heat (35, 38, 56), green space access and proximity (35–38, 56), and physical activity (36, 38, 56), of the population of interest. In all cases, access, exposure, proximity, and health effects in relation to the opera were examined (35–38, 56). Five studies assessed the health impacts of urbanization and transport infrastructure projects, including new transport planning (49, 55), new road (41, 51, 55, 58) and bridges (42) construction, and cycling and pedestrian paths (58), as well as the use of land (41, 55) and public facilities (41). Access (41, 49, 51, 55, 58), exposure (42, 49, 51, 58), proximity (41, 42, 51, 58), and health effects (41, 42, 49, 51, 55, 58) were assessed considering the specific opera.

Concerning urban planning-related exposure, 5 studies assessed the health impact of redeveloped or redevelopment areas (40, 43, 48, 50, 57), new processing plant (46), green space, and agriculture systems access and proximity (39), and urban energy transition initiative (47) on the surrounding communities. Access (39, 43, 47, 48, 50, 57), exposure (40, 46), proximity (43, 46–48, 57), and health effects (39, 40, 43, 46–48, 50, 57) opera-related have been explored.

Regarding industrial facilities, except for the one included in urban planning (46), only applications in the mining sector have been identified. Different types of industrial mining sites were examined, with gold mines being the most frequently studied (44, 45, 53, 59), alongside coal (44, 45, 59), ruby (44, 45, 59), titanium and manganese (45), copper (54), and uranium (52) mines. All studies considered factors such as exposure, proximity, and health effects on local communities and the workforce (44, 45, 52–54, 59). Additionally, one study specifically examined gender-related differences in access to mining sites (53).

#### 3.3.2 Inequities, inequalities, and vulnerabilities evaluated or to evaluate

In the included studies several key themes emerge around inequalities, inequities, and vulnerabilities concerning health impacts within specific socioeconomic and demographic groups, as well as to environmental factors that contribute to health disparities.

Following the Oxford Reference definitions (29–31) the following results have been observed: 10 studies assessed inequalities (35–38, 45, 49, 56–59), 11 studies evaluated inequities (40, 41, 44, 46–48, 50–54), and 4 studies faced inequities and vulnerabilities (39, 42, 43, 55).

Five studies (35–38, 56) showed that inequalities, influenced by socioeconomic factors, in exposure to air (35–38, 56) and noise pollution (36, 38, 56), green spaces (35–38, 56), heat (35, 38, 56), and physical activity (36, 38, 56), have been correlated with varying impacts on mortality.

Five studies (45, 49, 57–59) faced the potential inequalities linked to different impacts on health determinants (49, 59), and health indicators (57), focused on the potential difference in perceived health impacts by the affected communities (45, 58), considering also the possible health benefits for vulnerable populations (58).

Inequities and vulnerabilities emerged in 4 field studies (39, 42, 43, 55). Two of them (39, 55) evaluated the potential differences in accessibility to road and lift (55), and green spaces (39, 55) for vulnerable populations, including older adults, children, individuals with disabilities, those without parental support, and those with socioeconomic difficulties (39, 55). Two studies (42, 43), examined the perceived impacts of urban environment, health, quality of life, and health inequality issues, as reported by stakeholders (43) and community groups (42, 43). Perceptions were influenced by gender, age, and socioeconomic factors (43), as well as the proximity of vulnerable populations to the area affected by the work under study (42).

Inequities were assessed in 11 studies (40, 41, 44, 46–48, 50–54). Five studies (40, 44, 50, 53, 54) addressed potentially different impacts on health determinants perceived by the affected communities, according to socioeconomic factors (40, 44, 50), considering various subgroups within the affected population (men/women, adolescent boys/girls) (53), and conducting comparisons with analogous communities (54). Two studies (41, 52) assessed inequities related to the impact of public facilities, road transportation, and land use on low-income neighborhoods (41), and the impact of the proposed new park (48, 51) and road (51) on vulnerable people.

The different impacts of political and business practices, working conditions, and social, environmental, and economic conditions on workers and local communities (46, 52) were evaluated in two studies. Furthermore, the different impacts on vulnerable populations due to the energetic transition policy were assessed in one study (47).

### 3.4 Methods for assessing inequities, inequalities, and vulnerabilities in health impact assessment of new projects

This section offers a comprehensive overview of the qualitative and quantitative methodologies employed to analyze inequalities, inequities, and vulnerabilities within the HIA framework. Further in-depth information, is available in Supplementary File 2.

#### 3.4.1 Urban and transport planning

Inequalities as differential impacts of mortality due to environmental exposures faced in the cities, related to urban and transport planning, are closely linked to socioeconomic factors, as demonstrated by several studies (35–38, 56). These studies compared baseline exposure levels—such as air pollution (PM2.5, NO2) (35–38, 56), noise pollution (36, 38, 56), heat (35, 38, 56), green space access and proximity (35–38, 56), and physical activity (36, 38, 56)—with WHO-recommended exposure levels, referred to as “counterfactual scenarios.” To quantify the association between these exposures and mortality, exposure-response functions (ERFs) from the literature were applied, and relative risks (RRs) were scaled to reflect the differences between baseline and counterfactual exposure levels. Population-attributable fractions (PAFs) were then calculated to estimate the proportion of mortality attributable to these exposure level differences (35–38, 56).

To assess inequalities, studies used various measures of socioeconomic status (SES) as proxies, such as household income (37), and levels of deprivation (36, 56), encompassing frequently levels of education and employment/unemployment (35, 36, 38, 56). These were aggregated into SES indices (38), with stratified analyses conducted to explore disparities in exposure and mortality rates.

The Index of Multiple Deprivation (IMD) was used to classify residential areas by deprivation across dimensions like income, employment, education, and access to services (36). Additionally, in this case, the analyses were stratified by the proportion of non-White residents in the area involved in the project (36). A similar deprivation index, based on annual mean income per person percentage of the population without education, unemployment rate, and percentage of immigrants from low-and middle-income countries, was used to assess the association between SES, exposure levels, and attributable mortality rate (56).

In a further study, the Paulista Index of Social Vulnerability (IPVS) was employed to map socioeconomic vulnerability by different dimensions of poverty (e.g., income, education, life cycle) and spatial segregation in the cities of project (35), considering household income per capita, median income of female household heads, percentage of households with income below half and one-fourth of the minimum wage, literacy rate of household heads, percentage of household heads aged 10 to 29 years, percentage of female household heads aged 10 to 29 years, median age of household heads, and percentage of children aged 0 to 5 years (35).

Potential inequities, vulnerabilities, and health impacts from urban planning and transport interventions are reflected in different effects on disadvantaged groups, such as families without cars (51), residents in low-income neighborhoods (41), and the vulnerable such as older people and disabled (42, 55). Various methodologies were employed to capture these impacts, starting from literature reviews (41, 55), identifying health determinants and the potential effect of the project under consideration on them. After identifying specific health determinants, the potential influence of the project on these factors was evaluated in relation to the socioeconomic index, represented by housing prices, which were significantly correlated with income (41). Specifically, the assessment focused on potential impacts on the proportion of green space, per capita green space and its accessibility, service coverage of educational facilities, accessibility to recreational, healthcare, and commercial facilities, as well as the density of the road network, intersections, and land use (41). Moreover, qualitative data on access to green spaces, healthcare facilities, and transportation were collected through interviews and discussion groups with key informants and community members, allowing to identify key health impacts and potential differences between them, and formulating recommendations for improving the interventions (55).

In one study, three discussion meetings were held to assess the risks of the proposed road and park projects, examining health determinants such as the risk of road traffic accidents, traffic reduction/exclusion from residential roads, employment, changed outlook, property devaluation, and noise (51). The process involved community representatives, local health authorities, and council officers, and included a scoring system to evaluate health risks across different population groups, focusing on already vulnerable ones (51). Another study used 2 surveys, over 3 years, to collect baseline health, economic, and social data from residents, ensuring community involvement throughout (42). These surveys, administered face-to-face and via Computer Assisted Personal Interviews (CAPI), aimed to assess the health impacts of infrastructure projects, namely, the construction of a bridge, on population and vulnerable populations, particularly the younger and the older ones, by comparing responses across time and geographic areas, considering the different health impact of the project in relation to the distance from it (42).

To evaluate potential inequalities arising from the differential impacts of transport policies and planning (49, 58), including the implementation of new cycling and walking path projects (58), a literature and document review was conducted on the health determinants affecting vulnerable populations. This review was carried out by representatives of the local community (49, 58) and a multidisciplinary expert group (58) comprising transport planners, health board members, public health professionals, and community representatives. Drawing on evidence from the WHO, the expert group explored the connections between transport and health, identifying specific at-risk groups based on the city’s demographic profile (49). The group subsequently developed a methodology for assessing the risks posed to both vulnerable and non-vulnerable populations under three proposed transport planning scenarios, assigning scores ranging from −2 to +2 to each health determinant, such as accidents, pollution, physical activity, access to goods and services, and community networks (49). Furthermore, one-hour semi-structured interviews with stakeholders were conducted to collect their perspectives on the HIA project and process, confirm their participation, explore their specific interests in the target plan and HIA, and gather general impressions of the overall HIA approach (58). These were complemented by qualitative content analyses to evaluate the extent to which community and stakeholder concerns were incorporated into the final health impact assessments and related planning decisions (58).

#### 3.4.2 Urban planning

A range of qualitative and quantitative methods were used across different studies to assess inequities, inequalities, and vulnerabilities within HIA in the urban planning framework.

Reviews of the literature and document analysis were employed to assess the broader evidence base on health inequalities and the well-being of local populations potentially impacted by the projects (43, 46, 48). The application of the Merseyside Guidelines was recommended to identify health inequities within the HIA framework (50).

Interviews were commonly employed to identify the vulnerable, and assess community awareness of opera (39, 50), as well as community perspective (43). To analyze opinions of opera, considering strong or weak points and suggesting improvements, and also to evaluate changes in patterns of use of the opera, and perceived effects on health, an innovative mixed method called “Guerrilla Ethnography,” combining ethnographic observation and semi-structured interviews, was used (40). This method allowed for an in-depth analysis of perceived changes in social dynamics, use of public spaces, and overall health conditions, with particular attention to differences based on age, gender, and socio-economic status (40). Data collection involves multimedia tools (audio, video, photographs) and combines static and traveling observations with short-term individual or group interviews, emphasizing the physical context as a key element of analysis (40).

Furthermore, key informant interviews and individual meetings with stakeholders and also stakeholders workshops helped identify health concerns, such as those related to pollution (46), and the differential impacts on various community groups, enabling participants to review and refine related recommendations (43, 47, 48).

Surveys were another critical method, used to evaluate potential different access to green space, fresh food, as well as physical activity, social interactions, and skill development opportunities (39). Surveys investigated also the effects on mental and physical well-being (39, 40), social support (40), as well as the utilization of health services and unmet health needs (39) opera-related.

Furthermore, telephone surveys were used to gather perspectives from residents, with participants stratified by age, sex, and deprivation index, ensuring a representative assessment of health inequities (43).

Focus groups stratified by age, social class, and activity level provided rich qualitative data on stakeholders and community perspectives (43). This approach facilitated active community engagement in the evaluation process by enabling stakeholders to express their perspectives on municipal projects and their impacts, as well as to propose potential improvements. Moreover, it generated valuable insights into the interrelations between socio-historical, urban, and health dimensions. Data collection included recording and transcribing sessions with participants’ consent, and the subsequent analysis was conducted using a sociological discourse analysis framework (43). Discussions involving different demographic groups such as seniors and youth, as well as fathers or mothers of children living in or near the opera, the stakeholders involved in urban development and public health policy helped capture community-level perceptions of health impacts about environmental and social changes (40, 43, 57). One study utilized focus groups comprising over 50 participants from diverse populations and interest groups, which were instrumental in refining the HIA scope to 35 context-specific indicators and formulating research questions based on the project’s potential impacts (57). The importance of tailoring the analysis to the specific context and experiences of the affected population, thereby ensuring a nuanced understanding of local dynamics and impacts, is evident in another qualitative study that utilized focus groups to explore the perceptions of individuals living, studying, or working in the area under investigation, as well as the anticipated effects of the project (40). Each session, comprising 6 to 8 participants, involved 60 to 90 min of moderated discussion with the support of an observer (40). Similarly, focus groups explored the effects of public space use, mobility, social cohesion, and economic well-being in different involved groups (40). Generally, in all the studies (40, 43, 57), focus groups were employed to identify the health determinants that could potentially be impacted by the project, with particular attention to the various demographic groups under consideration. Additionally, another study (50) also suggests the use of focus groups as qualitative methods to identify inequities in HIA framework.

A study (46) also employed geospatial techniques, such as GIS mapping, to analyze environmental hazards and their health impacts on vulnerable populations, providing a spatial dimension to health inequities by identifying which communities were most affected by environmental risks.

These diverse methods enabled a comprehensive assessment of health inequities, inequalities, and vulnerabilities in HIA, capturing both subjective perceptions and objective health outcomes across different populations.

#### 3.4.3 Industrial mining site

Inequalities and inequities within HIA at industrial mining sites were assessed with a mixed-methods approach, integrating both qualitative and quantitative data collection techniques. In all studies, the HIA was conducted concurrently and encompassed various types of active mining operations, thereby facilitating a comprehensive evaluation of ongoing industrial activities.

Local data collection was undertaken as a preparatory stage, providing a fundamental understanding of the context (44, 45, 53, 59). Furthermore, a review of relevant documents and company literature was used to ensure a comprehensive assessment of the setting (52).

“Transect walks,” a guided tour through study sites led by local informants allowing researchers to observe, inquire, and gain a geographical and social overview of the area, were used to systematically identify communities positively and negatively affected by mining activities, such as those experiencing environmental degradation or community development (44, 45, 53). These walks facilitated the recruitment of participants for Focus Group Discussions (FGDs), which gathered qualitative data on social, cultural, economic, ecological, and political dimensions of health (44, 45). FGDs were conducted in 2 cases (44, 53) in gender-separated groups to promote open communication and to minimize gender-based power relations that might impede participants from talking freely. All FGDs were moderated by trained facilitators proficient in the local languages (44, 45, 53, 59). A participatory tool was employed during these discussions to collect, categorize, and rank impacts on the broader determinants of health (44, 45, 53), with discussions audio-recorded and transcribed for subsequent analysis (53, 59).

Additionally, key informant interviews (52, 59) and stakeholder meetings with policymakers (59) provided insights into the broader policy context and the impact of the opera on surrounding communities. Furthermore, in-depth interviews with impacted communities (52, 59) and semi-structured exit interviews at healthcare facilities focused on the perceived health impacts and the economic burden of diseases (59). In one case, interviews were designed to gather insights on health impacts identified through the Corporative-HIA framework, ensuring alignment with the study’s objectives (52). Potential respondents received detailed project information, invitations to participate, and consent forms via email. A total of 11 interviews were conducted remotely through telephone or Skype, facilitating accessibility and participation. To ensure accuracy and reliability, all interviews were professionally transcribed, supporting a thorough analysis of the collected data (52).

A study (54), involving 3 survey modules in the time spine of 3 years to collect data from women of reproductive age (15–49 years) on household characteristics and health-related practices, health indicators such as height, weight, and malaria infection in children under 5 years and women, and intestinal parasites and schistosomiasis infection in school children, assessed inequities in various health determinants and outcomes between the affected communities (9) and the comparison communities (4).

This comprehensive approach allowed for the identification and deeper understanding of the inequalities (45, 59), and inequities (44, 52–54) about health effects (44, 45, 52–54, 59), exposure (44, 45, 52–54, 59), access (53), proximity (44, 45, 52–54, 59), opera-related, experienced by different groups.

#### 3.4.4 IRIS—WHO guidelines and guidance

WHO guidance and guidelines highlight the need to address inequities, inequalities, and vulnerabilities through a nuanced and multi-methodological approach that combines both qualitative and quantitative techniques to capture the complex dynamics influencing health outcomes among different population groups (60–67). Frequently review of literature, which synthesizes existing evidence on environmental, health determinants, health, and social impacts of remediation and redevelopment of contaminated sites (62–65), provided a critical baseline, identifying populations at risk and laying the groundwork for understanding how different social, economic, and environmental factors may exacerbate health inequalities and inequities.

Qualitative methods play a crucial role in capturing the lived experiences and perceptions of specific project-affected populations. Interviews with key stakeholders—such as public health professionals, community leaders, and residents— as well as public consultations, workshops, and meetings could be employed to gather in-depth insights into local health concerns, especially those that disproportionately affect marginalized groups (60, 62, 64, 67). These interviews could be complemented by focus group discussions, and allow for an interactive discussion, which often reveals nuanced insights into how health interventions or policies may differently affect various segments of the population (67). These methods allow community members, particularly those who are often marginalized, to actively contribute to the assessment and propose solutions that may mitigate the negative health impacts of a given project or policy.

To provide a broader quantitative dimension, enabling the collection of data on specific health outcomes, surveys, and questionnaires were used (63, 66, 67). In 2015 and 2021 two surveys were distributed to the members of the European Environment and Health Task Force (EHTF), and an almost identical survey was distributed to experts on HIA—practitioners and academics—containing additional questions on the background of the respondents and the opportunity to relate answers to a country, region or municipality, to assess the status of inclusion of health in environmental assessments and equity consideration (63).

Furthermore, on the quantitative side, epidemiological models considering data on exposure are widely employed to assess the health impacts of environmental hazards (62, 64, 65, 67). Changes in air quality and noise levels during construction or operational phases of a project are quantified to estimate the potential health risks to nearby communities, particularly those already experiencing higher baseline levels of vulnerability due to socio-economic factors (62). These quantitative assessments help provide measurable evidence of how different interventions or project designs may impact health outcomes, often focusing on populations that are more sensitive to environmental changes due to pre-existing conditions or socioeconomic disadvantages (62, 64, 65, 67).

Additionally, geographical information system mapping, as an important tool that introduces a spatial analysis to HIA, helped describe the target population that is likely to be exposed to the hazard (60, 64), but also facilitated the stakeholder’s identification (61). By mapping environmental risks such as pollution or proximity to industrial sites, geographical systems enable the identification of communities that are disproportionately exposed to health risks.

Another prominent method is the Environmental and Social Impact Assessment (ESIA), which could be integrated into HIA frameworks to assess not only the physical health impacts of projects (such as air, water, and noise quality) but also the broader socio-cultural determinants of health. ESIAs take into account intangible and subjective health determinants, such as social cohesion, cultural identity, and economic well-being, offering a more holistic approach to understanding how vulnerable populations might experience the impacts of large-scale projects differently from the general population (62).

### 3.5 Main findings on inequities, inequalities, and vulnerabilities

This section presents the primary results concerning inequality, inequity, and vulnerability concerning the opera setting, as identified through the application of various methods in the HIA. Additional detailed information is provided in the Supplementary File 2.

#### 3.5.1 Urban and transport planning

Field studies across various cities highlight the differential mortality impacts of environmental exposures due to urban and transport planning, based on socioeconomic factors, with distinct patterns emerging for pollutants such as NO2, PM2.5, heat, green space, and noise (35–38, 56).

NO2 concentrations and related mortality varied across cities. In São Paulo, NO2 concentrations were higher in more socioeconomically vulnerable census tracts (CTs), with attributable mortality rates ranging from 30 deaths per 100,000 persons in the most vulnerable areas to 62 deaths per 100,000 in the least vulnerable (35). In Vienna, lower SES sub-districts experienced higher NO2 exposure, resulting in a mortality rate of 58.5 deaths per 100,000 persons, while no such association was found in higher SES districts (38). In Madrid, contrary to most other pollutants, NO2-related mortality was lower in the most deprived neighborhoods, where the attributable mortality rate was 51% lower compared to the least deprived quintiles In Barcelona, NO2 did not show a significant association with socioeconomic deprivation (56).

In Bradford, the most deprived residents experienced 11.5 deaths per 100,000 persons attributable to PM2.5 exposure, while the least deprived had nearly no related deaths (0.11 deaths per 100,000) (36). Consistent with this, the findings indicate that in Barcelona, the most deprived areas saw a 1.22 times higher mortality rate due to PM2.5 (56). In Madrid, PM2.5-related mortality was 1.86 times higher in the most deprived neighborhoods compared to the least deprived (56).

The impact of heat on mortality is highly heterogeneous, with rates of 3 deaths per 100,000 persons in highly vulnerable areas and 4 deaths per 100,000 in the least vulnerable in São Paulo (35), while in Vienna, lower SES districts had more severe heat-related mortality, with a rate of 18.3 deaths per 100,000 persons (38). Furthermore, in Barcelona, the most deprived CTs had a 1.27 times greater mortality risk due to heat exposure compared to the least deprived (56). In Madrid, no significant differences in heat-related mortality were observed across socioeconomic groups, indicating a more uniform impact of heat throughout the city (56).

Access to green space and its impact on mortality were significant across all cities. In São Paulo, areas with both high and very low socioeconomic vulnerability showed the highest green space levels-measured by the Normalized Difference Vegetation Index (NDVI)—but mortality rates ranged from 17, in socioeconomically unclassifiable, to 37, in the least vulnerable, deaths per 100,000 persons (35). In Vienna, lower SES districts, with limited access to green space, faced a mortality rate of 12.3 deaths per 100,000 persons (38), while in Bradford, deprived neighborhoods suffered from a significant lack of green space, contributing to 9.7 deaths per 100,000 persons, and the least deprived areas had almost no attributable deaths (36). In Barcelona, deprived areas had a 1.42 times greater mortality risk due to insufficient green space, and in Madrid, all deprivation quintiles had elevated mortality risks due to green space deficiencies compared to the least deprived areas (56).

Noise exposure also had a significant impact on mortality, especially in lower socioeconomic areas. In Vienna, lower SES districts faced higher noise exposure, leading to 3.5 deaths per 100,000 persons, while no such correlations were found in higher SES districts (38). In Bradford, noise exposure contributed to 10.27 deaths per 100,000 persons in the most deprived areas, whereas the least deprived areas experienced no attributable mortality (36). In Madrid, noise-related mortality was 20% lower in the highly deprived quintile compared to the least deprived group, while noise exposure in Barcelona did not show a significant association with socioeconomic deprivation (56).

A USA study highlighted significant health disparities linked to residential proximity to high-traffic roadways, particularly in socioeconomically disadvantaged areas (42). Individuals residing within 500 feet of the high-traffic roadway under examination and the surrounding area exhibit higher rates of asthma and respiratory allergies across all age groups, with the most pronounced effects observed in children under 5 and adults over 65 (42). The prevalence of asthma in children under 5 is 9.8%, compared to 4.6% in those living further from traffic, while for older adults, it is 24.4% compared to 12.9% (42). Furthermore, child health outcomes in these areas are notably worse, with infant mortality rates of 11.8%, compared to 5.1 to 8.8% in more distant areas (42). Residents living close to high-traffic zones are also more likely to consider relocating, likely due to perceived health risks and the poorer living conditions associated with air pollution (42). Related to high-traffic roads, the I-710 Corridor project in California encountered significant community opposition to the proposed highway expansion, which was deemed unacceptable by the surrounding area’s predominantly Latino and low-income residents due to health impacts (58).

In the Clark County Bicycle and Pedestrian HIA, several deficiencies in the planning process were identified, including a lack of facilities for inexperienced cyclists, and insufficient attention to low-income neighborhoods, suggesting potential inequities in the proposed improvements, despite the consideration of the potential positive effects associated with increased opportunities for physical activity (58).

In contrast, the Lake Merritt Bay Area Rapid Transit Station HIA effectively integrated social determinants of health framework, guided by community-defined principles that emphasize pedestrian safety, health pathways, social cohesion, employment opportunities for current residents, and the need for affordable housing to address gentrification concerns (58).

Four guidance documents (41, 49, 51, 55)—two from the UK (49, 51), one from Spain (55), and one from China (41)—emphasize the critical role of transport policy in addressing inequities (51, 62), inequalities (49), and inequities and vulnerabilities (55), albeit with differing approaches. In the context of urban transport reorganization, measures such as reducing private car usage and promoting cycling, walking, and public transport could offer substantial benefits to disadvantaged groups, who are disproportionately affected by traffic accidents, pollution exposure, limited physical activity opportunities, and restricted access to essential services (49). These groups stand to benefit most from healthier and more equitable transportation alternatives (49).

The Spanish guidance (55), in line with the Chinese ones (41), supports the potential positive outcomes of urban regeneration projects, such as improved access to healthcare and social networks, through infrastructure development, including communication systems (e.g., elevators and lifts) (55), new roads (41, 55), redevelopment of green spaces and land use (41, 55), and development of public facilities (41). These efforts could be especially beneficial for vulnerable populations, promoting physical activity and facilitating better access to services (41, 55), including educative ones (41). However, low-income neighborhoods within the older parts of the city may face increased challenges in accessing healthcare services and may no longer benefit from the proximity to commercial facilities that they previously enjoyed before the expansion (41). The Spanish guidance also warns of potential adverse effects, including exposure to pollution, increased risk of accidents, and greater access to alcohol and drugs near these green spaces (55).

Furthermore, the second UK guidance (51) points to the possible negative consequences of infrastructure projects, such as new roads, for disadvantaged families, particularly those without access to cars. These projects may limit access to natural amenities, such as national parks, thus reducing their potential contribution to well-being (51). Nonetheless, the same guidance acknowledges the potential health benefits of regenerating green spaces, although these benefits may be less accessible to disadvantaged families due to transportation barriers (51).

#### 3.5.2 Urban planning

Inequities and vulnerabilities are evident in the effects of urban planning and inaction on deprived areas (39, 43). Increasing green spaces and pedestrian areas can promote physical activity, social interaction, and access to services, particularly benefiting low-income individuals, the unemployed, pedestrians, cyclists, and vulnerable groups such as women, children, and the older adult (43). Survey data from Canada shows that 88% of those who depend on community green spaces for fresh food had an income of less than $15,000 Canadian, highlighting the link between poverty and reduced access to essential resources (39). Furthermore, urban areas and traffic-heavy zones face persistent challenges, including increased noise, pollution, and accident risks, disproportionately affecting vulnerable populations (43). In addition, inaction exacerbates problems such as deprived environments, poor land use, and unsafe public transport, reducing physical activity, and social cohesion, and, on the other side, increasing isolation (43). Vulnerable groups, particularly women, ethnic minorities, and low-income families, face increased insecurity, reduced access to services, and a decline in their sense of belonging and trust in institutions (43).

In the guidance addressing urban regeneration and energy transition initiatives, it has been noted that individuals with low incomes and those from socioeconomically disadvantaged backgrounds may be further disadvantaged by these initiatives, both in terms of inequity (47, 50) and inequalities (57). One of the English guidance highlights that residents in rental accommodations, likely with lower incomes, would find it challenging to gain financial benefits from the grant-assisted solar photovoltaic installations proposed under the energy transition initiative, as eligibility for grants is limited to homeowners (47). Additionally, these renters may face higher electricity costs through meter schemes (47).

Another English guideline suggests that small-scale regeneration initiatives may create a first scenario in which the majority of the current population remains, leading to an increase in average income, a reduction in unemployment, and an overall improvement in health in the area (50). However, this scenario may also result in increased disparities and decreased social cohesion. In a second scenario, where no improvements are anticipated or where deterioration occurs, some residents may benefit sufficiently to relocate, leaving behind the most disadvantaged (50). This could lead to a population shift, with newcomers facing similar socioeconomic challenges as those who remained. Furthermore, certain equity issues may be overlooked in the framing of policies or programs, resulting in the failure to address questions of inequality within the target population or between this population and other groups upon implementation. Worse still, inequalities within and between populations may even be exacerbated by regeneration initiatives (50).

In one of the USA guidance, the impact of various scenarios from the City Building and Future Growth Regeneration Initiative on health indicators was examined (57). The analysis found that sprawling development, which increases vehicle miles traveled (VMT), would render resources inaccessible to approximately 30% of the population who do not drive, including seniors, youth, low-income residents, and individuals with disabilities (57). Moreover, higher VMT also raises driving costs, disproportionately affecting low-income families (57). Regarding the percentage of households within a half-mile of a public elementary school, considerations of health disparities indicated that very rural populations, including Native American tribes and others, are unlikely to experience changes in their proximity to schools (57).

The Spanish guidance which places the”Superblocks” model at the center of urban reorganization, emphasizes how potential improvements in air quality, noise reduction, the distribution and availability of green spaces, mitigation of urban heat islands, transport density, and levels of physical activity could be linked to multiple health benefits (40). These include reductions in cardiovascular and respiratory diseases, depression, anxiety, and road accidents, as well as enhancements in social well-being, all of which could be differently influenced by socioeconomic factors (40).

Korean guidance indicated that in the project aimed at transforming a reservoir into a water park and central element of the city’s green space system, a segment of the population identified as vulnerable (including individuals with disabilities, low-income groups, and the older adult) may face challenges in accessing the park (48). As a result, they may not fully benefit from the anticipated positive outcomes of the project, such as exposure to a less polluted environment, opportunities for community engagement and social networking, increased physical activity, and improved access to healthcare and social services due to enhanced transportation systems resulting from the area’s redevelopment (48).

Another USA guidance on the conversion of a former pickle plant into a poultry processing facility found that the population residing in the affected area, of which one-fifth live below the federal poverty level, along with the plant workers, may be exposed to different levels of emissions associated with poultry processing, transportation, traffic, waste discharge, and odors (46). These exposures are expected to result in distinct health impacts compared to populations not affected by the facility (46).

#### 3.5.3 Industrial mining site

Perceived inequities in health determinants were identified across various subpopulation groups (men, women, adolescent boys and girls, and children) (53), and generally in communities surrounding industrial mining sites (44, 52, 54). These inequities encompassed a wide range of factors, including personal and community resources, living environments, soil and land conditions, water and air quality, access to healthcare facilities, and opportunities for income generation (44, 53). The construction and operation of mines have led to both positive and negative changes in local communities, contributing to increased perceived inequalities (44, 53). On the positive side, there are more employment opportunities, although these are unevenly distributed between and within communities (44). However, negative impacts include increased livelihood insecurity, reduced socioeconomic status, and hindered efforts to achieve good health and well-being, particularly due to environmental degradation caused by mining (44). While health services have improved, overall health opportunities are limited, particularly for children and adolescents, who are more vulnerable to the health impacts of mining (44). Furthermore, company policies influence health equity in complex ways: while supply chain practices have a positive impact on worker health, the influence of company policies can negatively contribute to health equity (52). Poor and low-paid working conditions incentivize unsafe work practices, with workplace fatalities in the South African mining industry being four times higher than in Australia (52). Additionally, noise and air pollution from mining is linked to psychological distress and increased risk of lung cancer; workers are exposed to dust and radon gas, and communities near coal rail corridors face increased risks of respiratory and cardiovascular disease (52). Despite these problems, there is a commitment to sustainable development principles, including restoring mine sites to vital ecosystems and monitoring emissions (52). It is noteworthy that in the study conducted in Zambia (54), communities affected by the mining project demonstrated improved health outcomes compared to control communities. This suggests that the health interventions implemented as a result of the HIA have successfully mitigated potential negative effects while enhancing positive outcomes. However, caution is warranted to prevent the inadvertent promotion of health inequalities both within the project area and in surrounding regions (54).

Women, whose roles include caregiving, domestic work, and secondary income generation, face greater challenges due to environmental degradation, which impacts their agricultural and domestic responsibilities (53). Increased illnesses among children due to mining activities have further limited women’s time for paid work (53). Despite improvements in health care, the burden on women has intensified, as they have primary responsibility for childcare. While men are responsible for the financial and physical support of their families, they often struggle to perform difficult and poorly paid mining jobs and have limited participation in child rearing, further exacerbating the pressure on women to balance caregiving and income generation (53).

In one guidance (59), however, measuring perceived inequalities in health determinants is planned for future assessment, providing an opportunity for more comprehensive data collection and analysis. These analyses will combine geographic factors with health outcomes and determinants in districts with and without extraction projects (59). Assessing the distribution of potential positive and negative impacts among various population subgroups—characterized by differences in gender, age, power, and occupational background—is essential to minimize inequalities in sustainable development (45), but, if the HIA conducted in mining areas has focused primarily on affected communities, as well as in Burkina Faso, Mozambique, Ghana, Tanzania guidance, without using comparison sites, this may introduce potential biases that disproportionately emphasize negative outcome, complicating the accurate prediction of future health impacts (45).

## 4 Discussion

This systematic review has provided insights into how inequalities, inequities, and vulnerabilities are addressed within HIA procedures, particularly in relation to local environmental risks and benefits. The findings highlight significant variability in the methodologies used across different sectors, such as urban and transport planning, and industrial mining, which reflects the complexity of assessing health equity (68) in diverse settings. Even in studies conducted within the same context, including those on mining sites (44, 45, 52–54, 59), mixed methods are employed, integrating common components that do not fully overlap. Consequently, no systematic methodology is defined for application, even when the setting and type of opera are the same. Adaptation to the context may be guided by the investigators’ choices or specific requirements imposed by the context itself.

One of the key findings is the consistent association between SES and differential health outcomes. Evans et al. proposed “*multiple risk exposure*” as a critical mechanism in the SES and health gradient, whereby individuals in lower SES brackets experience an accumulation of adverse physical and psychosocial factors (69). These compounded exposures likely intensify health risks, contributing significantly to health disparities observed across SES levels (69). The use of SES, operationalized through composite indices (35, 36, 56) or factors, such as average annual income (37, 39, 48), housing ownership costs (41), and rental living (47), has facilitated a more nuanced stratification of impacted populations. This methodology proves particularly effective in urban settings, characterized by the coexistence of individuals with disparate economic conditions. In such contexts, stratification grounded in economic criteria enables the identification of varied impacts stemming from developmental initiatives. Nevertheless, in addition to economic stratification, demographic and social vulnerability criteria have also been employed, particularly age (39, 42, 43, 55), disabilities (39, 55), chronic diseases (39, 42), or gender (43), albeit in a subordinate capacity.

In accordance with the principles of Health in All Policies (HiAP), most guidelines advocate for the involvement of affected communities in HIAs (70). This involvement is crucial for capturing local perspectives and health concerns through qualitative data collection methods, including focus groups, surveys, and interviews (39, 40, 42, 43, 46, 48–51, 55, 57, 58). Effective community participation necessitates clearly defined methodologies (71). As noted by Elliott et al. (72), public engagement should encompass not only participation in decision-making processes but also the critical scrutiny of expert claims. This approach underscores that inclusion extends beyond mere consultation to actively questioning expert perspectives. However, methodologies for the practical systematic implementation of community values in HIAs have yet to be established, indicating a gap in the operationalization of these principles.

While some transport policies and urban redevelopment projects are capable of yielding benefits for vulnerable populations (39–41, 43, 48–50, 55), it is crucial to recognize the potential negative effects, including increased pollution and reduced access to essential services (35–37, 41–43, 46–48, 50, 51, 55, 57, 58).

Conversely, in mining sites, a contrasting dynamic is evident. These areas are predominantly populated by economically disadvantaged communities, rendering SES stratification based on economic criteria less efficacious apparently. In this context, demographic vulnerability has emerged as the primary stratification criterion, with particular emphasis on groups such as the older adult, children, women, and male mine workers, who are most susceptible to the hazards associated with mining operations (44, 45, 52–54, 59). A recent systematic review (73) underscores the necessity of integrating the perspectives of these groups into methodologies for evaluating the socioeconomic impacts of mining.

The economic component of SES is subsequently considered, reflecting the relative economic homogeneity of these regions, with attention to employment status and income (44, 45, 52, 53). These populations were not only more vulnerable to environmental degradation but also faced increased health risks due to occupational hazards and limited access to healthcare. On the other side, results of this review showed that communities affected by the mining project demonstrated improved health outcomes compared to control communities, suggesting that health interventions stemming from the HIA mitigated potential harms and enhanced benefits (54).

This dual approach facilitates a more precise assessment of the impacts of developmental projects across varying contexts, thereby addressing the specific needs inherent to each locality.

Assessment techniques within HIA are increasingly addressing equity issues, also largely influenced by numerous recommendations from the WHO, as emerged from results of the present review (60–67). HIA itself originates from three interconnected areas of public health: environmental health, the wider social determinants of health, and health equity (74). Many HIAs claim to incorporate equity considerations but often conflate this with an analysis of social determinants, merely categorizing populations by their level of vulnerability (75). This review indicates a shift towards HIAs that account for broader health implications stemming from social, political, and economic inequities. This evolution highlights a growing awareness of the structural determinants underpinning health disparities and the need for more comprehensive and methodologically robust approaches. Such approaches should be grounded in multidisciplinarity and interdisciplinarity, alongside active engagement with stakeholders and communities (76).

### 4.1 Strengths and limitations

This systematic review exhibits several strengths that enhance its rigor and reliability.

By adhering to the PRISMA guidelines (26), the review ensures a systematic and transparent approach, minimizing bias and increasing reproducibility. Furthermore, the application of the “snowballing” method aids in identifying pertinent studies through references, ensuring a more exhaustive literature coverage (27). The integration of grey literature, particularly through the WHO’s IRIS (28), enriches the review with practical insights that may not be captured in traditional academic publications.

Furthermore, the utilization of standardized definitions for key concepts as well as “*Inequalities, Inequities, and Vulnerabilities*” alongside the PROGRESS plus framework for assessing equity-related variables guarantees consistency and rigor in the analysis (29–32). It is essential to recognize that these terms should not be used interchangeably, given their distinct meanings, consistently with existing literature (77). Failing to adequately distinguish between inequalities, inequities, and vulnerabilities can result in analyses that neglect the underlying structural causes of health disparities. This oversight could diminish the effectiveness of policy interventions designed to promote health equity, as it risks addressing symptoms rather than the root causes of injustice in health outcomes.

Reporting data according to the nature of opera (e.g., urban and transport planning, mining sites) enhances the understanding of differences among studies and aids in identifying specific trends, thereby improving the ability to interpret and apply the findings.

By highlighting the variability in methodologies, even when studies are conducted within the same settings and on similar types of opera, this review synthesizing the existing evidence could serve as a starting point for the creation and development of a common, unified framework for future HIA, considering the equity dimension. Establishing minimum standards that must consistently be met at each stage of the HIA process is recommended, irrespective of the context or type of project under evaluation. The use of standardized tools and instruments, such as the one employed in this study-Wales Health Impact Assessment Quality Assurance Review Framework (34) in our case used to assess how many included works addressed equity and how they did so-is beneficial for the methodological consistence of HIA. However, such tools necessitate precise responses to predefined criteria and methodological accuracy, which, as our findings indicate, are often insufficiently addressed in the existing literature—not only with regard to equity but across several aspects of HIA practice.

However, the review is not without limitations. The exclusive focus on English-language publications may overlook significant contributions from non-English studies, potentially limiting the comprehensiveness of the assessment on available methods. Furthermore, we acknowledge that the study predominantly includes research from high-income countries, but this could stem from a true lack of studies conducted in low-and middle-income countries or from the language limitations of our search. Moreover, It is also possible that in developing countries, such evaluations are conducted for different types of projects that were not captured by our search strategy, or that while HIAs may be performed, the studies are not published in peer-reviewed literature, contributing to the apparent scarcity of research from these regions. On the other hand, including the WHO’s IRIS (28), has enabled us to assess and incorporate documents that would likely have been missed, highlighting the valuable insights that can be found in this source. This suggests that grey literature, often underutilized, may contain important findings not captured in traditional academic publications. Nevertheless, it is worth noting that all eight studies included from the WHO IRIS focus on developed countries. Given this limitation, it could be beneficial for future research to explore additional grey literature databases to uncover more evidence related to developing countries.

The absence of temporal restrictions on the literature search could lead to the inclusion of outdated studies that may not reflect current practices.

Furthermore, the methodological quality assessment relies on the Wales Health Impact Assessment Quality Assurance Review Framework (34), which, while providing a systematic evaluation, may be subject to varying interpretations among reviewers. While this framework is a valuable tool for assessing the quality of HIA, including considerations of equity, the limited number of specific and general questions on equity reveals that much remains to be done in this area. This is particularly evident as responses to these questions often indicate that equity is still inadequately and insufficiently evaluated within HIA practices. Even among HIAs that fulfilled the majority of the tool’s requirements adequately or more than adequately, equity considerations were not always sufficiently addressed. Conversely, instances were observed where equity was emphasized despite overall lower adherence to other tool criteria, highlighting a persistent inconsistency in the integration of equity within HIA practices. It should also be noted that HIAs rarely consider health in its entirety (39, 40, 43), encompassing not only physical but also mental well-being. Furthermore, no clear patterns emerged linking types of operations or methodologies to higher quality or to a health impact assessment that can be called comprehensive in all respects. This highlights existing gaps that need to be addressed to achieve a more holistic assessment of health within HIA practices. Moreover, although only a few studies explicitly reported conducting a rapid HIA (46, 47, 49) which does not typically include a monitoring phase, almost all reviewed assessments lacked a clear monitoring methodology. Without systematic monitoring, it becomes challenging to assess the long-term health and equity impacts of interventions, track progress over time, or identify unintended consequences. Furthermore, this absence limits the ability to establish evidence-based feedback loops, refine methodologies, and share lessons learned. This gap also inhibits the dissemination of best practices and actionable insights, reducing opportunities for scaling successful interventions or improving equity integration. Furthermore, the majority of studies failed to identify specific indicators, qualitative or quantitative, to track and evaluate the health impacts over time. As our findings illustrate, HIA studies frequently prioritize qualitative and participatory approaches to capture local perspectives and stakeholder input. Another limitation identified in our systematic review is the lack of studies employing quantitative methods, such as exposure-response functions or GIS-based analyses. This gap may be attributed to the complexity and resource demands of these methods, which require comprehensive datasets, specialized technical expertise, and interdisciplinary collaboration. Additionally, the use of such methods is often constrained by limited data availability and the context-specific nature of many HIA studies, which tend to emphasize qualitative methods to better capture the distinctive characteristics of the local context. Despite these limitations, the strengths of this systematic review provide a robust foundation for understanding the status of the art in addressing health inequalities, inequities, and vulnerabilities in the context of health impact assessments regarding local projects.

As well known, the HIA process is structured into distinct phases, each with clearly defined objectives to ensure a comprehensive and evidence-based evaluation of health impacts (78). The screening phase aims to determine whether an HIA is necessary by assessing the potential health relevance of a given policy, program, or project. This phase relies on decision-support tools, such as screening checklists, to evaluate the likelihood of significant health impacts. The scoping phase establishes the scope and objectives of the HIA, delineating its boundaries, identifying relevant health determinants, and prioritizing affected populations. Stakeholder engagement and conceptual frameworks are critical tools in this phase to ensure inclusivity and alignment with policy goals (78, 79).

The assessment phase serves as the core analytical component, where potential health impacts are systematically identified, predicted, and analyzed (78). This involves the use of both qualitative and quantitative methodologies, such as risk assessment models, epidemiological data analysis, and participatory methods, to evaluate the nature and magnitude of health effects (78, 79). In the recommendations phase, evidence-based strategies are developed to enhance positive impacts and mitigate negative ones, utilizing decision matrices and multi-criteria analysis to ensure actionable and feasible guidance (78).

The reporting phase focuses on disseminating findings and recommendations to stakeholders and decision-makers, often employing clear, structured reporting templates to enhance transparency and usability (78, 79). Finally, the monitoring and evaluation phase tracks the implementation of recommendations and assesses their effectiveness, contributing to iterative learning and improving future HIA practices (78, 79). Tools such as performance indicators and outcome evaluation frameworks are essential in this phase to ensure accountability and measure success.

The methods identified in this review provide valuable insights into enhancing the equity and comprehensiveness of HIA processes, particularly during the screening, scoping, and assessment phases. For screening, the use of literature and document reviews allows for an early identification of health risks and opportunities, offering a structured approach to determining the need for an HIA, also for identifying sub-groups or categories of the local populations with pre-existing fragilities and vulnerabilities.

During the scoping phase, tools such as policy analysis, focus groups, interviews, and transect walks demonstrate significant potential to ensure inclusivity and tailor the HIA to the equity dimension. These methods help identify vulnerable populations (with more reliability than in the screening phase) and context-specific health determinants. Nevertheless, they require substantial time and resources and may not fully capture all relevant factors, posing challenges for resource-constrained settings.

In the assessment phase, the integration of surveys, interviews, comparisons of exposure levels with counterfactual scenarios, and GIS methods (with their power to provide evidence on the spatial distribution of different risk and beneficial factors and of different population groups) provides a robust framework for predicting and analyzing health impacts and has the potential for assessing environmental health inequalities. These methods offer a comprehensive view of potential outcomes, facilitating evidence-based decision-making. However, limitations such as data availability and the subjective nature of participatory approaches must be addressed to improve reliability and applicability in diverse contexts.

Collectively, these methods underscore the importance of incorporating tailored, context-sensitive tools to address health inequalities and inequities effectively, while highlighting the need to balance methodological rigor with practical feasibility.

### 4.2 A practical framework for integrating equity into HIA

Building on this established framework and based on our findings, we propose an operational approach to practically integrate the equity dimension into HIA. Specifically, we suggest incorporating the methods identified in our analysis into the phases where they are most appropriate, depending on their contribution to the HIA process, as shown in the Table 3. This practical framework aims to support practitioners in selecting and applying the most suitable methods for each phase, ensuring a comprehensive and equity-driven HIA process.

**Table 3:** Comparison between established methods and the methods identified in review, along with their strengths and limitations, to guide the integration of equity in HIA.

HIA Step	Objectives	General Methods in Literature (78, 79)	Methods derived from the Review	Strengths	Limitations
Screening	Determine if HIA is necessary for the project	Screening checklists, health impact scoring	Literature and relevant documents review and analysis	Enhances early identification of health risks and opportunities	Requires clear criteria; may miss nuanced impacts
Scoping	Define scope, objectives, health determinants, and affected populations	Stakeholder consultation, conceptual frameworks	Policies analysis, Focus group, Meeting, Interview, Transect walk	Ensures inclusivity, tailored approach to equity	Can be time and resources related, may not capture all relevant factors
Assessment	Identify, predict, and analyze potential health impacts	Epidemiological analysis, risk models, qualitative analysis	Survey, Interview, Comparison of exposure levels and counterfactual scenarios, GIS	Provides a comprehensive view of health impacts	Data limitations, subjectivity in participatory methods
Recommendations	Develop strategies to enhance positive impacts and mitigate negative ones	Decision matrices, multi-criteria analysis, expert judgment	Equity-driven prioritization, targeted interventions for vulnerable groups	Promotes equitable decision-making, actionable strategies	Implementation challenges, resource constraints
Reporting	Communicate findings and recommendations to stakeholders	Structured reporting templates, policy briefs	Transparent reporting with equity considerations, accessible formats	Increases clarity, transparency, and stakeholder engagement	Risk of oversimplification, needs for broader stakeholder input
Monitoring and Evaluation	Track implementation and assess the effectiveness of recommendations	Performance indicators, outcome evaluation frameworks	Equity-focused monitoring, tracking health disparities	Ensures accountability, fosters continuous improvement	Requires long-term commitment, data availability issues

**Figure 3:**
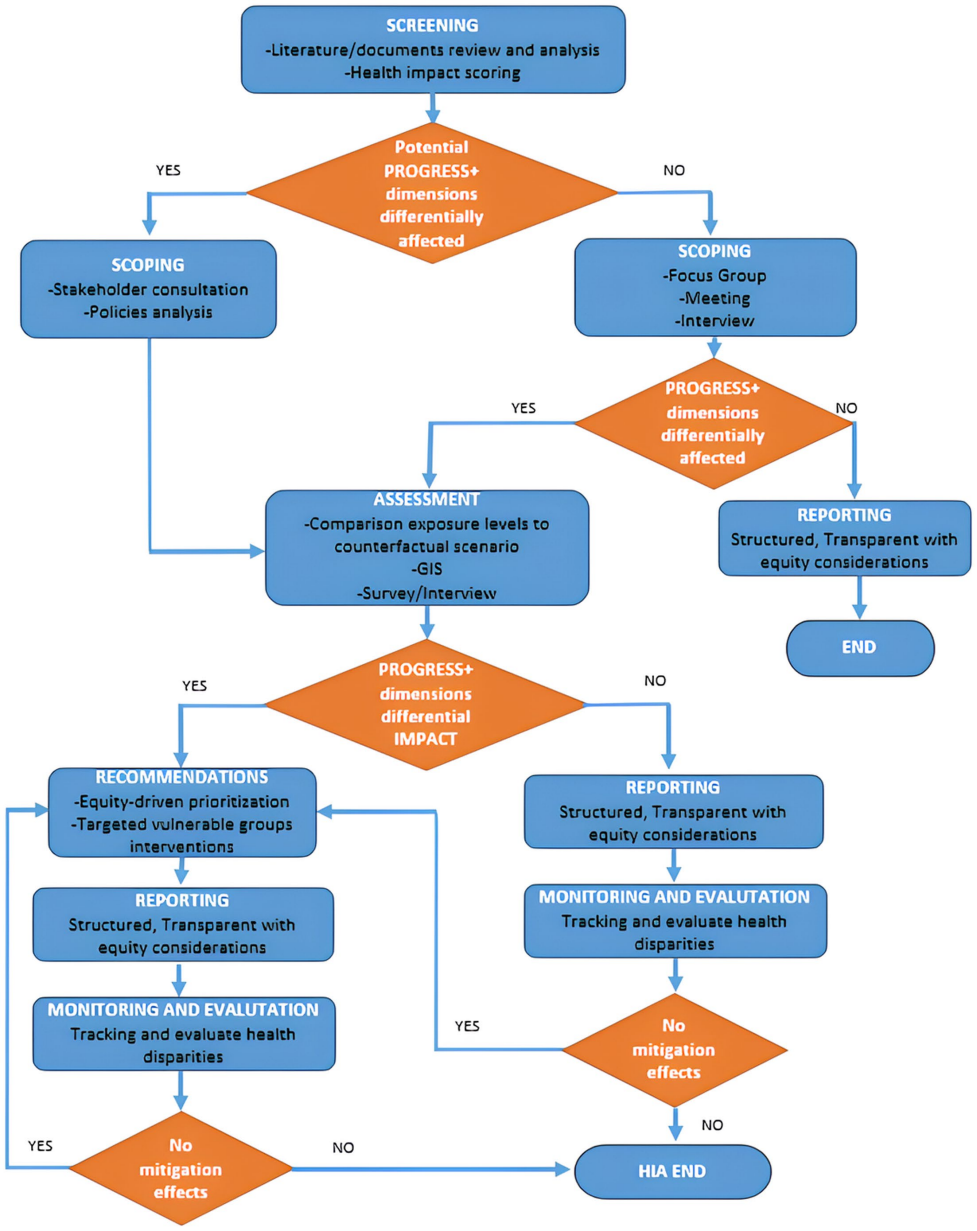
Decision-making flowchart for addressing inequalities in HIA.

The Figure 3 outlines a structured process for integrating equity considerations into HIA, so helping in taking decisions and choosing methods depending on results on inequalities/inequities in each HIA phase. The process begins with a *Screening* phase, where literature review and health impact scoring are conducted to determinate whether specific PROGRESS+ dimensions are differentially affected.

If the *Screening* phase indicates that PROGRESS+ dimensions are potentially impacted, the process moves to *Scoping*, involving stakeholder consultations and policy analysis to further refine the scope, objectives, health determinants, and affected populations. However, if the screening does not initially identify a differential impact, the *Scoping* phase may instead employ methods that directly engage potentially affected populations—such as focus groups, meetings, or interviews—to conduct a more in-depth exploration and verify whether disparities may still exist. If no significant impact is confirmed at this stage, the process concludes with *Reporting*.

The *Assessment* phase may vary depending on the preceding scoping process. If the scoping phase engaged directly with affected populations, the assessment is more likely to rely on qualitative methods, as the information gathered stems from direct interaction with communities, often through interviews, narratives, and participatory approaches. Conversely, if the scoping phase was based on stakeholder consultations and policy analysis, the assessment is more likely to use quantitative methods, such as comparison of exposure levels, counterfactual scenario modeling, and GIS-based spatial analysis.

Following the *Assessment* phase, if a differential impact on PROGRESS+ dimensions is identified, the process advances to the *Recommendations* stage. This phase emphasizes equity-driven prioritization, ensuring that proposed interventions specifically target the most vulnerable groups. Recommendations are formulated based on the nature and extent of inequities, inequalities and vulnerabilities observed during the assessment, guiding the development of mitigation strategies.

Conversely, if the *Assessment* phase does not identify a differential impact on the PROGRESS+ dimensions, the process advances directly to the *Reporting* phase, omitting the *Recommendations* stage. The *Reporting* phase entails a structured and transparent process, incorporating explicit equity considerations to document findings, proposed actions, and anticipated outcomes. This ensures accountability and facilitates stakeholder engagement in decision-making.

The process then transitions to *Monitoring and Evaluation*, which involves systematically tracking health disparities over time. This step assesses the effectiveness of implemented measures and ensures that the proposed interventions yield tangible improvements in equity outcomes. If *Monitoring and Evaluation* reveal persistent differential impacts, the process loops back to *Recommendations*. Conversely, if no further equity-related disparities are detected, the HIA process is concluded.

This structured approach reinforces an iterative, evidence-based decision-making process that integrates equity at each stage.

## 5 Conclusion

In conclusion, this systematic review has elucidated the complexities surrounding the assessment of health inequalities, inequities, and vulnerabilities within HIAs regarding local projects. The findings reveal significant variability in methodologies employed across different sectors, such as urban planning and industrial mining, underscoring the multifaceted nature of health equity assessments. While there is a consistent association between SES and health outcomes, the review emphasizes the importance of context-specific adaptations in HIA methodologies to effectively address the unique challenges faced by diverse populations.

Furthermore, this review highlights the critical role of community involvement in HIAs, advocating for the integration of local perspectives and health concerns through qualitative methods. Despite the increasing recognition of equity within HIAs, there remains a notable gap in systematic methodologies that facilitate the practical implementation of community values. This gap indicates a need for further research to develop robust frameworks that can consistently incorporate diverse community voices and address structural determinants of health. The available evidence highlights the importance of developing these frameworks, combining qualitative and quantitative methods through interdisciplinary work.

## Data Availability

The original contributions presented in the study are included in the article/Supplementary material, further inquiries can be directed to the corresponding author.
