# Supplementary material for: Exploring methods to assess environmental health inequalities in health impact assessments of local interventions: a systematic review within the JA PreventNCD project
Source: Front Public Health. 2025 Mar 19;13:1546394. doi: 10.3389/fpubh.2025.1546394 (PMC11961916; doi:10.3389/fpubh.2025.1546394)
Supplement: Supplementary file 1 [file Data_Sheet_1.docx]

| **Search engine** | **Search strategy** |
| --- | --- |
| PubMed/MEDLINE | From 1000/01/01 to 08/03/2024  ("health impact assessment"[MeSH Terms] OR "health impact assessment"[Text Word] OR "health equity impact assessment" OR "HIA") AND ("inequities"[All Fields] OR "inequity"[All Fields] OR "inequalities"[All Fields] OR "inequality"[All Fields] OR "unequal"[All Fields] OR "environmental justice"[MeSH Terms] OR "environmental justice"[Text Word] OR "environmental injustice" OR "vulnerable"[All Fields] OR "vulnerabilities"[All Fields] OR "marginalization"[All Fields] OR "marginalized"[All Fields] OR "vulnerable populations"[MeSH Terms] OR "disadvantaged"[Text Word] OR "disadvantages"[All Fields]) AND ("environment"[MeSH Terms] OR "environment"[Text Word] OR "parks, recreational"[MeSH Terms] OR "green space"[Text Word] OR "industrial site" OR ("contaminated"[All Fields] AND "site"[All Fields]) OR "city planning"[MeSH Terms] OR "city planning"[Text Word] OR "infrastructure"[All Fields]) |
| Scopus | At 08 March 2024  (TITLE-ABS-KEY(health impact assessment) OR TITLE-ABS-KEY(health equity impact assessment) OR TITLE-ABS-KEY(HIA)) AND (TITLE-ABS-KEY(inequities) OR TITLE-ABS-KEY(inequity) OR TITLE-ABS-KEY(inequalities) OR TITLE-ABS-KEY(inequality) OR TITLE-ABS-KEY(unequal) OR TITLE-ABS-KEY(environmental justice) OR TITLE-ABS-KEY(environmental injustice) OR TITLE-ABS-KEY(vulnerable) OR TITLE-ABS-KEY(vulnerabilities) OR TITLE-ABS-KEY(marginalization) OR TITLE-ABS-KEY(marginalized) OR TITLE-ABS-KEY(vulnerable populations) OR TITLE-ABS-KEY(disadvantaged) OR TITLE-ABS-KEY(disadvantages)) AND (TITLE-ABS-KEY(environment) OR TITLE-ABS-KEY(parks, recreational) OR TITLE-ABS-KEY(green space) OR TITLE-ABS-KEY(industrial site) OR (TITLE-ABS-KEY(contaminated) AND TITLE-ABS-KEY(site)) OR TITLE-ABS-KEY(city planning) OR TITLE-ABS-KEY(infrastructure)) |
| Embase | At 08/03/2024  ('health impact assessment' OR 'hia' OR 'health equity impact assessment') AND ('inequities' OR 'inequity' OR 'inequalities' OR 'inequality' OR 'unequal' OR 'environmental justice' OR 'environmental injustice' OR 'vulnerable' OR 'vulnerabilities' OR 'marginalization' OR 'marginalized' OR 'vulnerable populations' OR 'disadvantaged' OR 'disadvantages') AND ('environment' OR 'parks, recreational' OR 'green space' OR 'industrial site' OR ('contaminated' AND 'site') OR 'city planning' OR 'infrastructure') |

**Table 1**- Search strategy for scientific literature

| **First Author, year** | **Title** | **Exclusion Rationale** |
| --- | --- | --- |
| Yoo W., 2007 | Introduction of health impact assessment and healthy cities as a tool for tackling health inequality | Full text in original language |
| Barral S., 2021 | Development of two tools for improving consideration of environmental health and multi-exposure situations in Paris | No full text |
| Prochaska J., 2012 | Health inequities in environmental justice communities: Relevant indicators to reflect a variety of health threats | No full text |
| Xia T., 2022 | Black carbon exposures and trends in Detroit, Michigan: Synthesis of fixed, mobile and indoor measurements | No full text |

**Table 2**- Records not retrieved from databases

| **First Author, year** | **Title** | **Exclusion Rationale** |
| --- | --- | --- |
| Anguelovski I., 2021 | Gentrification pathways and their health impacts on historically marginalized residents in Europe and North America: Global qualitative evidence from 14 cities | Wrong outcome |
| Bailey J., 2019 | Potential health and equity co-benefits related to the mitigation policies reducing air pollution from residential wood burning in Athens, Greece | Wrong outcome |
| Bhatia R., 2008 | Integrating human health into environmental impact assessment: An unrealized opportunity for environmental health and justice | Wrong outcome |
| Buregeja J.M., 2019 | Contribution to healthy places: Risks of equity free health impact assessment | Wrong outcome |
| Douglas M., 2001 | Addressing health inequalities in health impact assessment | Wrong outcome |
| Douglas MJ., 2001 | Achieving better health through health impact assessment | Wrong outcome |
| Douglas MJ., 2001 | Developing principles for health impact assessment | Wrong outcome |
| Khreis H., 2019 | The health impacts of urban transport: Linkages, tools and research needs | Wrong outcome |
| Leuenberger A., 2021 | Health impacts of industrial mining on surrounding communities: Local perspectives from three sub-Saharan African countries | Wrong outcome |
| Mehdipanah R., 2019 | Effects of Superblocks on health and health inequities: A proposed evaluation framework | Wrong outcome |
| Olyaeemanesh A., 2023 | Health Equity Impact Assessment (HEIA) reporting tool: developing a checklist for policymakers | Wrong outcome |
| Pasetto R., 2023 | Environmental justice promotion in the Italian contaminated sites through the national epidemiological surveillance system | Wrong outcome |
| Ritsatakis A., 2013 | Equity and the social determinants of health in European cities | Wrong outcome |
| Solomon G.M., 2016 | Cumulative Environmental Impacts: Science and Policy to Protect Communities | Wrong outcome |
| Storm I., 2015 | The relevance of work-related learning for vulnerable groups. Dutch case study of a Health Impact Assessment with equity focus | Wrong outcome |
| Bretagne G., 2022 | Tackling social inequalities in health: acceptability and feasibility of a systematic approach toward health impact assessment of urban projects. | No HIA |
| Babagoli M., 2019 | Exploring the Health and Spatial Equity Implications of the New York City Bike Share System. | No HIA |
| Clari C., 2020 | Longitudinal impact of changes in the residential built environment on physical activity: Findings from the ENABLE London cohort study | No HIA |
| Hong A., 2021 | Neighbourhood green space and health disparities in the global South: Evidence from Cali, Colombia | No HIA |
| Iroz-Elardo N., 2020 | Active travel and social justice: Addressing disparities and promoting health equity through a novel approach to Regional Transportation Planning | No HIA |
| Krieg E.J., 2004 | Not so Black and White: Environmental justice and cumulative impact assessments | No HIA |
| Pottie K., 2019 | HEIA tools: inclusion of migrants in health policy in Canada | No HIA |
| Randal E., 2022 | The Impact of Transport on Population Health and Health Equity for Māori in Aotearoa New Zealand: A Prospective Burden of Disease Study | No HIA |
| Winters M., 2018 | Impacts of Bicycle Infrastructure in Mid-Sized Cities (IBIMS): Protocol for a natural experiment study in three Canadian cities | No HIA |

**Table 3-** Full texts excluded **from databases**

| **First Author, year** | **Title** | **Exclusion Rationale** |
| --- | --- | --- |
| Esnaola S., 2010 | La evaluación del impacto en la salud: una vía para introducir la salud en todas las políticas. Informe SESPAS 2010 | Full text in original language |
| Venegas-Sánchez J., 2012 | Evaluación del impacto en la salud del proyecto de reurbanización de la calle San Fernando en Alcalá de Guadaíra (Sevilla) | Full text in original language |
| Iroz-Elardo N., 2014 | Health impact assessment as community participation | Duplicate |
| Khomenko S., 2020 | Is a liveable city a healthy city? Health impacts of urban and transport planning in Vienna, Austria | Already included from databases |
| Mueller N., 2018 | Socioeconomic inequalities in urban and transport planning related exposures and mortality: A health impact assessment study for Bradford, UK | Already included from databases |
| Pereira-Barboza E., 2022 | The impact of urban environmental exposures on health: An assessment of the attributable mortality burden in Sao Paulo city, Brazil | Already included from databases |

**Table 4-** Records not retrieved from **snowballing**

| **First Author, year** | **Title** | **Exclusion Rationale** |
| --- | --- | --- |
| Pereira-Barboza E., 2021 | Green space and mortality in European cities: a health impact assessment study | Wrong outcome |
| Hendryx M., 2015 | The public health impacts of surface coal mining | Wrong outcome |
| Hricko A., 2014 | Global trade, local impacts: lessons from California on health impacts and environmental justice concerns for residents living near freight rail yards | Wrong outcome |
| Leuenberger A., 2021 | Health impacts of industrial mining on surrounding communities: Local perspectives from three sub-Saharan African countries | Wrong outcome |
| Iungman T., 2023 | Cooling cities through urban green infrastructure: a health impact assessment of European cities | Wrong outcome |
| Khomenko S., 2022 | Impact of road traffic noise on annoyance and preventable mortality in European cities: A health impact assessment | Wrong outcome |
| Knoblauch Astrid M., 2017 | Monitoring of Selected Health Indicators in Children Living in a Copper Mine Development Area in Northwestern Zambia | Wrong outcome |
| Knoblauch Astrid M., 2018 | Selected indicators and determinants of women’s health in the vicinity of a copper mine development in northwestern Zambia | Wrong outcome |
| Mehdipanah R., 2019 | Effects of Superblocks on health and health inequities: a proposed evaluation framework | Wrong outcome |
| Mueller N., 2016 | Urban and Transport Planning Related Exposures and Mortality: A Health Impact Assessment for Cities | Wrong outcome |
| Mueller N., 2017 | Health impacts related to urban and transport planning: a burden of disease assessment | Wrong outcome |
| Mueller N., 2018 | Health impact assessment of cycling network expansions in European cities | Wrong outcome |
| Rojas-Rueda D., 2013 | Health impact assessment of increasing public transport and cycling use in Barcelona: A morbidity and burden of disease approach | Wrong outcome |
| Rojas-Rueda D., 2012 | Replacing car trips by increasing bike and public transport in the greater Barcelona metropolitan area: A health impact assessment study | Wrong outcome |
| Salcito K., 2015 | Experience and lessons from health impact assessment for human rights impact assessment | Wrong outcome |
| Maas J., 2006 | Green space, urbanity, and health: how strong is the relation? | No HIA |
| Nicholas Will., 2019 | Routine Assessment of Health Impacts of Local Transportation Plans: A Case Study From the City of Los Angeles | No HIA |
| Urman R., 2018 | Risk effects of near-roadway pollutants and asthma status on bronchitic symptoms in children | No HIA |
| Vert C., 2019 | Health Benefits of Physical Activity Related to an Urban Riverside Regeneration | No HIA |

**Table 5-** Full texts excluded **from snowballing**

| **First Author, year** | **Title** | **Exclusion Rationale** |
| --- | --- | --- |
| World Health Organization. Regional Office for Europe, 2023 | Setup, roles and tasks of a support unit: policy brief 2 on health impact assessments and incorporating health into environmental assessments | Wrong outcome |
| World Health Organization. Regional Office for Europe, 2023 | Generic guidelines and tools: policy brief 4 on health impact assessments and incorporating health into environmental assessments | Wrong outcome |
| World Health Organization. Regional Office for Europe, 2023 | Health impact assessment of steel plant activities in Taranto, Italy | Wrong outcome |
| World Health Organization. Regional Office for Europe, 2021 | Solid waste management and health in Accra, Ghana | Wrong outcome |
| World Health Organization. Regional Office for Europe, 2023 | Economics of the health implications of waste management in the context of a circular economy | No HIA |
| World Health Organization. Regional Office for Europe, 2015 | Chemicals of public health concern in the African Region and their management: Regional Assessment Report | No HIA |
| World Health Organization. Regional Office for Europe, 2013 | Cross-country analysis of the institutionalization of health impact assessment / Jennifer H. Lee, Nathalie Röbbel and Carlos Dora | No HIA |
| World Health Organization. Regional Office for Europe, 2019 | From linear to circular economy: health implications of sustainable consumption and production: report of WHO meeting: 12–13 November 2018, Bonn, Germany | No HIA |
| World Health Organization. Gibson G, Nowacki J, Cave B, 2013 | Capacity Building in Environment and Health (CBEH) project: strengthening health in environmental assessments in Slovenia: gap analysis and way forward | No HIA |
| World Health Organization. Gibson G, Nowacki J, Cave B, 2013 | Capacity Building in Environment and Health (CBEH) project: strengthening health in environmental assessments in Estonia: gap analysis and way forward | No HIA |
| World Health Organization. Regional Office for Europe, Health management and planning, 2013 | Opportunities for scaling up and strengthening the health-in-all-policies approach in South-eastern Europe | No HIA |

**Table 6-** Full texts excluded **from grey literature**

| **Reference** | **Opera Setting** | **Type of Assessment (Access, Exposure, Proximity, Health Effects, Use)** | **Inequities, inequalities, and vulnerabilities** | **Findings on inequities, inequalities, and vulnerabilities** |
| --- | --- | --- | --- | --- |
| E.P Barboza et al., 2022 | Urban and Transport Planning related exposure (green space, air pollution and heat) | Exposure, Health Effects | Inequalities Different mortality impacts of exposure by socioeconomic factors. | High' (vulnerable) and 'very, very low' (least vulnerable) socioeconomic vulnerability CT showed the highest NDVI (Normalized Difference Vegetation Index) with respectively 21 and 22 deaths/100,000 persons, while 'very low' and 'very high' showed the lowest, with respectively 37 and 28 deaths/100,000 persons. NO2 concentrations increased from the most socioeconomically vulnerable to the least vulnerable CTs, resulting in 34 deaths/100,000 persons in 'very high', and 62 deaths/100,000 persons in 'very, very low'. Heat mortality rate had a low variation of impact by socioeconomic vulnerability (respectively 4 and 3 deaths/100,000 persons in 'very, very low' and 'very high' CT). Across all exposures, CTs of ‘very low’ socioeconomic vulnerability had the largest environmental exposure-attributable mortality rate (95 deaths/100,000 persons), while CTs of ‘high’ socioeconomic vulnerability had the lowest (54 deaths/100,000 persons). |
| N.Mueller et al., 2018 | Urban and Transport Planning related exposure (Physical Activity (PA), air and noise pollution, and green space) | Access, Exposure, Health Effects | Inequalities Different mortality impacts of exposure by socioeconomic factors. | Stratified analyses by socioeconomic position (SEP) showed that residents of more deprived and more ethnically-diverse LSOAs (Lower Super Output Area level) were at higher risk for adverse environmental exposures and mortality. Most deprived residents showed 11.5 deaths/100,000 persons PM2.5 attributable, 10.27 deaths/100,000 persons noise attributable, 9.7 deaths/100,000 persons lack of green space attributable; least deprived residents showed 0.11, 0, and 0.33 deaths/100,000 persons respectively for PM2.5, noise, and lack of green space. |
| E.P Barboza et al., 2023 | Urban and Transport Planning related exposure (green space and air pollution) | Access, Exposure, Health Effects | Inequalities Different mortality impacts of exposure by socioeconomic factors. | Overall environmental exposures were correlated with the average annual income (strongest relationships in Umeå and Versailles). In Lahti, Tallinn, and Umeå the areas of lower income levels tended to have lower exposure to green spaces, higher exposure to air pollution, and higher attributable mortality impacts in comparison to areas with higher income levels.  In Limerick and Versailles the areas of lower in­come levels tended to have higher exposure to green space, lower exposure to air pollution, and lower mortality impacts in comparison to areas with higher income levels. |
| S. Khomenko et al., 2020 | Urban and Transport Planning related exposure (PA, air and noise pollution, green space and heat) | Access, Exposure, Health Effects | Inequalities Different mortality impacts of exposure by socioeconomic factors. | Lower socioeconomic status (SES) sub-district showed higher NO2, noise, and temperature exposures, and lower green space, with higher and significantly increased related-mortality rate (respectively 58.5 deaths/100,000 persons for NO2, 3.5 deaths/100,000 persons for noise, 18.3 deaths/100,000 persons for heat, 12.3 deaths/100,000 persons for green space). No such correlations were found for higher SES sub-districts. No differences were observed in the total natural-cause mortality rate by SES. |
| T.Iungman et al., 2021 | Urban and Transport Planning related exposure (PA, air and noise pollution, green space and heat) | Access, Exposure, Health Effects | Inequalities Different mortality impacts of exposure by socioeconomic factors. | In Barcelona, the most deprived areas had higher mortality rates due to PM2.5, lack of green spaces, and heat exposure, with mortality risks up to 1.42 times greater compared to the least deprived areas. NO2 and noise-related mortality did not show significant associations with deprivation. In Madrid, PM2.5-related mortality was also higher in deprived areas, while NO2 and noise-related mortality were lower. Lack of green space increased mortality across all deprivation levels, and heat exposure showed no significant difference between deprivation groups. |
| N. Sampson et al., 2020 | Urban and Transport Planning related exposure (Gordie Howe International Bridge) | Exposure, Proximity, Health Effects | Inequities and Vulnerabilities Different health impacts on vulnerable residents in the survey area; neighborhood perceptions, and intention to move from the area | Asthma prevalence is higher among people living within 500 feet of I-75 or trucking routes compared to those living farther away: <5 years old 9.8% (within 500 feet) vs. 4.6% (more than 500 feet); 5-17 years old 19% vs. 13.4%; 18-40 years old 9.1% vs. 6.6%; 41-64 years old 14.4% vs. 13.6%; 65 years and older 24.4% vs. 12.9%. Respiratory allergies or asthma rates are higher for those living closer to the roadways: <5 years old 18.3% (within 500 feet) vs. 10.6% (more than 500 feet); 5-17 years old 33% vs. 22.9%; 18-40 years old 25.5% vs. 20.9%; 41-64 years old 34.8% vs. 29.7%; 65 years and older 42.2% vs. 32.9%. Those living near heavily trafficked roadways showed increased disease risk (e.g. cardiovascular, COPD, mortality, compromised lung function, neurological). Infant mortality is 11.8% (deaths to infants <12 months), for the entire survey area and neighborhoods of Southwest Detroit, in contrast to 8.8 ± 3.6 in Detroit and 5.1 ± 0.3 in Michigan. The residents of the impact area were more likely to indicate that they planned to move within one year, or the next 1–5 years, compared to those living in the buffer area north of I-75. |
| Y. Ge et al., 2022 | Urban and Transport Planning related exposure (public facilities, road transportation and land use) | Access, Proximity, Health Effects | Inequities Potential impacts in low-income neighborhoods related to public and healthcare facilities, road transportation, and land use | Positive Impacts of Expansion on Low-Income Neighborhoods: Increased amount and accessibility of green space, educational facilities, service area, and recreational facilities; enhanced urban road network; improved residential environment and land use mix. Negative Impacts of Expansion on Low-Income Neighborhoods: Loss of accessibility to commercial facilities; inequities in accessibility to healthcare facilities in the old town. |
| V. Anderson et al., 2021 | Urban Planning related exposure (green space, and agriculture and tree-based intercropping systems) | Access, Health Effects, Use | Inequities and Vulnerabilities Potential impacts of green infrastructure on vulnerable populations (seniors,children, individuals of lower SES, individuals with chronic illness or disability) | Survey results showed that users of the green roof who did not have access to fresh outside of the Carrot Green Roof and Community Garden were members of vulnerable populations including those of lower income. 47% of the green roof users had no access to fresh food outside the Carrot Green Roof and Community Garden, and 88% of them had an annual income of less than $15k CAD. |
| L. Palència et al., 2020 | Urban Planning related exposure (Superblocks) | Exposure, Health Effects | Inequities Potential impacts perceived related to mental health and health determinants, according to socioeconomic factors. | Possible changes in traffic injuries, number of people and pedestrians injured, unequal reduction in cardiovascular and respiratory diseases, depression, and anxiety, and an enhancement in social well-being. |
| E. Serrano et al., 2016 | Urban Planning related exposure (New fish market (NFM) and redevelopment of the La Herrera (LH) zone) | Access, Exposure, Proximity, Health Effects | Inequities and Vulnerabilities Potential impacts perceived by stakeholders and community groups on the urban environment, health/quality of life and health inequality issues, according to gender, age, and socioeconomic factors. | NFM Increased green spaces, recreational areas, and pedestrian walkways enhance PA, social interaction, and access to services, benefiting the local population nearby. Increased noise, odors, pollution, and heavy traffic negatively impact the population, especially in nearby urban areas, increasing the risk of accidents for vulnerable groups (children, youths, and the elderly).  LH Effects of non-intervention Deteriorating environment affect the entire population, particularly vulnerable groups (women, elderly, children, and those with low incomes). Poor land use and unsafe access to the metro decrease PA and social cohesion, increasing isolation and accidents for the entire population particularly vulnerable groups. Worsening conflicts with sponsor institutions lead to increased mistrust, social frustration, and reduced community belonging and self-esteem, particularly affecting socially excluded individuals. |
| A. Leuenberger et al., 2021 | Industrial Mining Site related exposure | Exposure, Proximity, Health Effects | Inequities Perceived inequities, related to health determinants (personal factors and resources, social and community network , living environment and subsistence work , job opportunities and conditions, general socio-economic-political conditions) in interested communities | Positive changes: More job opportunities, even if not distributed equally among and within communities.  Negative changes: More insecure livelihoods and decreased socioeconomic status; achieving good health and well-being was hindered by the mine's construction and operation.  Children and adolescents are particularly vulnerable to the mining impacts on health. Health equity is worsening in the context of industrial mining projects. |
| J.Anaf et al., 2019 | Industrial Mining Site related exposure | Exposure, Proximity, Health Effects | Inequities Positive and negative impacts on workers and local communities related to political and business practices, workforce and working conditions, social conditions, environmental conditions, and economic conditions | Workers face more precarious, lower-paid conditions, leading to unsafe work practices. High wages for miners raise local prices, while an influx of migrant workers disrupts nearby communities. On the other hand, there is an increased local Aboriginal participation in the workforce, infrastructure, and local procurement support. Workers are exposed to dust and radon gas, air pollution, soil contamination; noise and air pollution from mining are associated with psychological distress, and increased risk of lung cancer; on the other hand there is a commitment to sustainable development principles and to restore the uranium mine area to a viable ecosystem, to monitor emissions and dust levels. Business strategies negatively impact revenue for social and health investment; on the other hand contribute to national and local economies. |
| A.Leuenberger et al., 2021 | Industrial Mining Site related exposure | Exposure, Access, Proximity, Health Effects | Inequities Difference in perception of impacts on the wider determinants of health, in subpopulation groups (men, women, adolescent boys and girls, and children). | Women's roles encompass being mothers and housewives, secondary income generators, and engaging in low-prestige community activities. Environmental degradation from mining particularly affects women's agricultural and domestic work. Women also bear the primary responsibility for childcare, which has become more demanding as children fall sick more often due to mining activities, reducing women's time for paid work. Improved healthcare services benefit women in their caregiving roles. Men are responsible for financial and physical household maintenance. Their involvement in child-rearing is limited, and they may struggle to meet financial expectations, increasing the burden on women. Jobs in the mines are difficult and low-paying, adding to the challenges faced by men in fulfilling their breadwinner role. |
| A.M. Knoblauch et al., 2020 | Industrial Mining Site related exposure | Exposure, Proximity, Health Effects | Inequities Different health determinants and outcomes in impacted communities versus the comparison communities | Communities impacted by the mining project frequently showed better health outcomes than comparison communities, suggesting that the health interventions implemented by the project as a consequence of the HIA have mitigated potential negative effects and enhanced positive effects. Caution is indicated to avoid the promotion of health inequalities within and beyond the project area. |

**Table 5-** Main Findings from Peer-Reviewed Field Studies

| **Reference** | **Opera Setting** | **Type of Assessment (Access, Exposure, Proximity, Health Effects, Use)** | **Inequities, inequalities, and vulnerabilities** | **Inequities, inequalities, and vulnerabilitiesc to be considered** |
| --- | --- | --- | --- | --- |
| L. Baskin-Graves et al., 2019 | Urban Planning related exposure (chicken processing plant) | Exposure, Proximity, Health Effects | Inequities Potential different impacts on vulnerable populations living nearby the area or working in the area | Exposures related to poultry processing, transport, waste discharge, odor issues, occupational health, and traffic concerns |
| J. Richardson et al., 2012 | Urban Planning related exposure (Transition Together/Transition Streets (TT/TS) projects) | Access, Proximity, Health Effects | Inequities Potential different impacts of energetic transition on vulnerable populations (low-income households, households in rented houses) | Identify the changes needed and the barriers to change to include the most disadvantaged groups in transition initiatives |
| E. Kang et al., 2011 | Urban Planning related exposure (Aegi-Neung Waterside Park) | Access, Proximity, Health Effects | Inequities Potential differences in access and use of green spaces among the vulnerable populations (disabled, lower-income people, and older people) | Affordable and accessible transportation options/systems for vulnerable groups |
| R. Barnes et al., 2002 | Urban Planning related exposure (Regeneration initiatives (New Deal for Communities (NDC), Single Regeneration Budget (SRB)) | Access, Health Effects | Inequalities Potential different impacts on health determinants | Regeneration initiatives improve the area but also increase inequalities or worsen the status quo, leaving the most disadvantaged behind and attracting similarly disadvantaged newcomers. |
| E. C. Harris et al., 2009 | Urban Planning related exposure (General Plan Update (GPU) to guide future building and growth) | Access, Proximity, Health Effects | Inequalities Potential different impacts on health indicators | Enhance walkability, bikability, and public transportation as health-promoting interventions, especially for non-driving populations (seniors, youth, low-income individuals, and disables) |
| D. Gorman et al., 2003 | Urban and Transport Planning related exposure (Transport planning) | Access, Exposure, Health Effects | Inequalities Potential different impacts on the health determinants on deprived groups (young families, adolescents, elderly, working people, and unemployed) | Disadvantaged groups are most impacted by the negative effects of economical transportation planning but gain the most from the positive outcomes of more comprehensive, cost-effective plans. |
| C. Lester et al., 2004 | Urban and Transport Planning related exposure (Proposed road; the area also includes a country park development) | Access, Exposure, Proximity, Health Effects | Inequities Potential different impact of proposed road and park on groups who were already disadvantaged (families without car) | Impacts of road development, including traffic accidents, noise, and property devaluation, but also employment opportunities |
| A.Bacigalupe et al., 2009 | Urabn and Transport Planning related exposure (Integral Regeneration Plan (IRP) of Uretamendi-Betolaza and Circunvalación (UBC)) | Access, Exposure, Health Effects | Inequities and Vulnerabilities Potential differences in accessibility to Lifts/roads, green spaces for vulnerable (older people, disabled, people without parents). | Lifts/Roads Strenghtening of social networks and social cohesion, promote walking, easier access to health centre, improvement of home visits by professionals, easier access to a variety of cheaper food, increase of traffic on roads and injuries near lift exit. Improvements in accessibility will have a lesser effect on the area of Betolaza in general and a greater effect on older people, the disabled, people without a car and parents. Grenn spaces, Park Better perception of neighborhood, increase of physical activity, and social networks, but also promotion of drug and alcohol consumption. The park will have a lesser effect on the area of Betolaza in general and a greater effect on low socioeconomic groups, the youth, older people and parents. |
| N. Iroz-Elardo et al. 2014 | Urban and Transport Planning related exposure (Clark County Bicycle and Pedestrian HIA, Lake Merritt BART Station Area Plan HIA, and the I-710 Corridor HIA) | Access, Exposure, Proximity, Health Effects | Inequalities Potential health benefits for vulnerable populations; consideration of community perspectives regarding safety and social cohesion, employment, and health | Assess potential impacts on access to physical activity; integrate health determinants with community perspectives (public safety, community facilities, transportation, cultural preservation, community engagement, jobs, and health.); examine the effects of highway expansion on the community |
| A.Farnham et al., 2020 | Industrial Mining Site | Exposure, Proximity, Health Effects | Inequalities Potential different impacts on health determinants | Lack or poor implementation of monitoring systems at mining sites which could lead to an underestimation of the incidence of disease and health impacts |
| A. Leuenberger et al., 2022 | Industrial Mining Site | Exposure, Proximity, Health Effects | Inequalities Potential different perceived health impacts by the surrounding communities | Considering the distribution of potential positive and negative impacts among population and population subgroups (characterized by different gender, age, occupational background) is particularly important to minimize inequities in sustainable development. The tools used focused on affected communities in mining areas, without comparison sites, potentially biasing results by emphasizing negative impacts. |

**Table 6-** Main Findings from Peer-Reviewed Guidance
