# Supplementary material for: Exploring methods to assess environmental health inequalities in health impact assessments of local interventions: a systematic review within the JA PreventNCD project
Source: Front Public Health. 2025 Mar 19;13:1546394. doi: 10.3389/fpubh.2025.1546394 (PMC11961916; doi:10.3389/fpubh.2025.1546394)
Supplement: Supplementary file 3 [file Data_Sheet_3.docx]

*Methodological quality assessment*

The methodological quality assessment was conducted using the Wales Health Impact Assessment Quality Assurance Review Framework, a standardized form of quality assurance for HIAs.

This tool enabled a systematic evaluation of the HIA process by focusing on key criteria such as stakeholder engagement, the integration of evidence, and the thoroughness of impact evaluations. The Wales Health Impact Assessment Quality Assurance Review Framework was originally designed to assess the comprehensiveness of the HIA approach rather than the quality of the study’s outcomes. Moreover, its validity is intended to apply on a case-by-case basis, as it was not developed as a comparative tool for evaluating multiple studies against one another.

The tool consists of two appendices: a primary appendix known as the Review Criteria Matrix and a supplementary appendix referred to as the Explanatory Notes. Appendix One is structured into six sections, each corresponding to a section in Appendix Two.

In total, the six sections comprise 43 questions, for each of which there is an answer that indicates the quality, such as "good", "requires strengthening”(this could be at three levels: Clarification needed, Minor revision needed, Major revision needed), or "insufficient".

Our objective in using the tool was to determine how many studies addressed equity-related questions and how they did so, while also gaining a broader understanding of the quality of HIA approaches in the examined studies. Since the tool does not define specific ranges for quality but relies on the knowledge and experience of researchers in HIA to assess the quality level, we limited ourselves to applying the tool and reporting the number of responses classified as "good", "requires strengthening", or "insufficient". To ensure consistency in the application of the tool, all assessments were carried out by the researchers involved with experience in HIA. In cases where there were disagreements in the assessment, these were resolved through collaborative discussion. The team reached consensus by revisiting the specific criteria and answers, guided by the framework’s explanatory notes and the collective expertise of the researchers involved.

If we were to provide a qualitative summary assessment for each study, though arbitrary and based solely on the knowledge and experience of the researchers applying the tool, we could consider a study "Good" if it received a "Good" response to at least 26 out of the 43 total questions, representing at least 60% of responses deemed comprehensive..

The equity dimension is primarily addressed in Section 6 titled “Principles and Governance: Has it been conducted in a way that meets the principles and values of HIA?“, which includes a specific and direct question on equity (*A focus on contributing to achieving equity and reducing inequalities is considered throughout the HIA*), along with four additional questions that focus on transparency (*The governance of the HIA is clear and appropriate to ensure that the HIA was carried out in an effective and balanced way*), democracy (*This emphasises the rights of people to participate in major decisions that affect their lives. The stakeholders engaged reflect the diversity of all those who are likely to be affected by the proposal, involved in the development of the proposal or involved in the implementation of the proposal*), sustainability (*The HIA set out to maximise health and wellbeing benefits/impacts and minimise unintended consequences by considering both short and long-term impacts*), and participation (*The HIA used appropriate, effective and accessible methods of engagement for the stakeholders who were relevant for this assessment)* within the HIA framework.

Additionally, another questions in Section 4, titled “Appraisal, assessment and the identification of impacts”, indirectly investigate the equity dimension, with a question about inequalities (*It is clear who will be impacted and any potential inequalities in the distribution of impacts are identified*).

By applying this framework, we ensured a comprehensive analysis of the HIA's methodological rigor and adherence to established best practices in the field.
